# Supplementary material for: Curvature-based machine-learning method for automated segmentation of dendritic spines
Source: Biophys J. 2026 Jun 5;125(14):3604–19. doi: 10.1016/j.bpj.2026.06.005 (PMC13335156; doi:10.1016/j.bpj.2026.06.005)
Supplement: Supplementary file 2 [file mmc2.pdf]

Biophysical Journal
Article

Biophysical Society

Curvature-based machine-learning method for automated segmentation of dendritic spines

Abdel Kader A. Geraldo,[1] Michael A. Chirillo,[2] Kristen M. Harris,[3] and Thomas G. Fai[4,*]
[1]Department of Mathematics, Brandeis University, Waltham, MA, USA; [2]Department of Biological Sciences, University of Rhode Island, Kingston, RI, USA; [3]Department of Neuroscience, University of Texas at Austin, Austin, TX, USA; and [4]Department of Mathematics and Volen Center for Complex Systems, Brandeis University, Waltham, MA, USA

ABSTRACT Recent advances in connectomics have been led by high-resolution reconstruction of large volumes of neural tissues using electron microscopy (EM), providing unprecedented insights into brain structure and function. Dendritic spines—dynamic protrusions on neuronal dendrites—play crucial roles in synaptic plasticity, influencing learning, memory, and various neurological disorders. However, current spine-analysis methods often rely on manual annotation of subcellular features, limiting their ability to handle the complexity of spines in dense dendritic networks. This paper introduces a novel automated computational framework that integrates discrete differential geometry, machine learning, and 3D image processing to analyze dendritic spines in these intricate environments. By generating distributions of spine morphology from high-resolution images including many thousands of spines, our approach captures subtle variations in spine shapes, offering a nuanced understanding of their roles in synaptic function. This framework is tested on multiple EM datasets with the aim of enhancing our understanding of synaptic plasticity and its alterations in disease states. The proposed method is poised to accelerate neuroscience research by providing a scalable, objective, and comprehensive solution for spine analysis, uncovering insights into the role of spine geometry for neural function.

SIGNIFICANCE We develop a deep-learning method to probe key aspects of the structure of neuronal dendritic spines and formulate a series of neural-network geometries that improve spine detection. The training data consist of manually segmented dendritic segments from hippocampal area CA1 of juvenile rats. We validate the method by applying the trained model to data obtained from published reconstructions of spiny dendrites and demonstrate that our method successfully learns key features of neuronal structure. By taking this step toward the automation of spine segmentation, we are able to obtain statistical information on individual synaptic structures and correlations between their geometries.

INTRODUCTION

Recent advances in electron microscopy (EM) and connectomics have made it possible to reconstruct astonishingly large volumes of neural tissue at nanometer resolution.[1,2] These breakthroughs have opened new opportunities for studying brain microstructures, particularly dendritic spines, dynamic protrusion on dendrites where most excitatory synapses in the brain occur. The morphology of dendritic spines—encompassing shape, size, and density—is highly plastic and closely tied to fundamental neurological processes such as learning and memory.[3–7]

Quantitative studies have shown that fine-scale geometric features of spines, including head volume, neck length, and curvature, are strongly associated with synaptic strength, developmental state, and functional specialization.[8–10] Changes in spine structure have also been linked to drug abuse, environmental influences, and a wide range of neurodevelopmental, neurodegenerative, and psychiatric disorders.[11–13]

Accurate detection and analysis of dendritic spines in three-dimensional (3D) reconstructions is therefore essential for advancing our understanding of synaptic organization and plasticity. However, spine identification remains challenging due to their heterogeneous morphologies, dense clustering, and local curvature variations. Manual annotation is highly time intensive and impractical for large-scale datasets, underscoring the need for automated and reliable computational methods. Several segmentation approaches

Submitted December 29, 2025, and accepted for publication June 2, 2026.
*Correspondence: tfai@brandeis.edu
Editor: Guoyou Huang.
https://doi.org/10.1016/j.bpj.2026.06.005
© 2026 The Author(s). Published by Elsevier Inc. on behalf of Biophysical Society.
This is an open access article under the CC BY license (http://creativecommons.org/licenses/by/4.0/).

have been proposed in recent years,[14–22] yet challenges remain in achieving both accuracy and scalability.

In particular, many existing 3D convolution neural-network (CNN)-based methods[23–25] operate directly on voxel representations, which require dense volumetric grids and therefore scale poorly to large EM datasets. This voxel dependence leads to substantial memory overhead, slow inference, and difficulty capturing fine geometric details such as thin spine necks. More broadly, large EM volumes contain millions of surface elements, creating significant computational constraints for voxel-level processing or repeated high-resolution inference. Together, these challenges underscore the need for automated approaches that incorporate geometric cues and remain robust across large, heterogeneous datasets.

To address these challenges, we introduce a method for automated spine segmentation that combines discrete differential geometry with deep learning. Our framework begins by preprocessing 3D reconstructions of dendritic segments to smooth the triangulated mesh, reduce noise, and enhance surface quality. From the resulting meshes, we extract geometric descriptors such as Gaussian and mean curvature along with additional features including distances from the dendritic shaft skeleton and clustering-based descriptors. Together, these features provide a rich representation of both local and global morphology.

Building on this geometric foundation, we developed a series of deep neural-network (DNN) architectures to evaluate how feature enrichment impacts segmentation performance. The baseline model, DNN_1, relies primarily on curvature-based descriptors. DNN_2 extends this by incorporating distance-to-skeleton features, improving the separation of spines from shafts. Finally, DNN_3 integrates a set of enriched geometric and topological descriptors, enabling the network to capture subtle morphological variations and complex spine arrangements. This progression of models demonstrates how systematically incorporating new features enhances both training convergence and segmentation accuracy.

In this work, we use deep learning to generate a dataset consisting of over 500 segmented spines and analyze the distributions of spine neck diameter, spine area, and spine volume along with their correlations. The resulting distributions address a fundamental biological question of how form and function are related in dendritic spines.[3,26]

MATERIALS AND METHODS

In this section, we employ differential geometry to design a DNN architecture for the segmentation of dendritic spines. As a first step, we analyze how curvature can contribute to the characterization of dendritic morphology. Building on this analysis, we then develop and explore a DNN that leverages the geometric properties of dendrites to achieve accurate segmentation. For convenience, a summary table of the symbols and variables used throughout the materials and methods section is provided in Table S1.

Dendritic morphology analysis using discrete differential geometry

Segmentation of dendritic shafts and spines can be guided by their differential geometric properties, particularly Gaussian and mean curvature. These curvature measures capture local shape variations that distinguish the roughly cylindrical shaft from protruding spines. However, raw curvature values obtained directly from EM reconstructions are often noisy due to the sectioning process and mesh irregularities. To address this, we first smooth the dendritic triangulated surface mesh using discrete differential-geometry techniques, then compute curvature values, and finally enhance these through image-processing filters. This three-step process—smoothing, curvature computation, and enhancement—provides robust geometric descriptors that form the basis for accurate segmentation.

Mean and Gaussian curvature

In this section, we motivate the use of Gaussian and mean-curvature values as key variables for segmenting the dendritic shaft. In the study of dendritic morphology, the shaft is typically modeled as an approximately cylindrical structure from which spines protrude. The discrete mean $\mathbf{H}$ and Gaussian curvatures $\mathbf{K}$ computation details are provided in Section S5.

The Gaussian curvature provides insight into the surface's shape at different points. At hyperbolic points, where the surface curves in opposite directions (saddle-like), the Gaussian curvature is negative. Conversely, at elliptical points, where the surface curves uniformly in the same direction (dome-like), the Gaussian curvature is positive. This contrasts with the cylindrical shaft, along which the Gaussian curvature is zero. Applying these principles to dendritic morphology, we observe that, along the spine neck and at the intersection between the neck of a spine and the dendritic shaft, the surface exhibits a saddle shape, leading to negative Gaussian curvature. On the other hand, the spine head, with its more spherical or dome-like structure, exhibits positive Gaussian curvature.

The mean curvature further characterizes the surface. On a concave surface, the mean curvature is positive, while, on a convex surface, it is negative. This distinction helps to identify regions of significant shape change. For instance, at the transition from the dendritic shaft to the spine neck, the surface is concave, leading to relatively positive mean curvature. In contrast, the spine head, which is a convex region, has relatively negative mean curvature.

These curvature measures are most useful for segmentation when the triangular mesh is sufficiently smooth, as smoothness preserves the overall geometry of the dendritic branches while reducing discretization noise. Under these

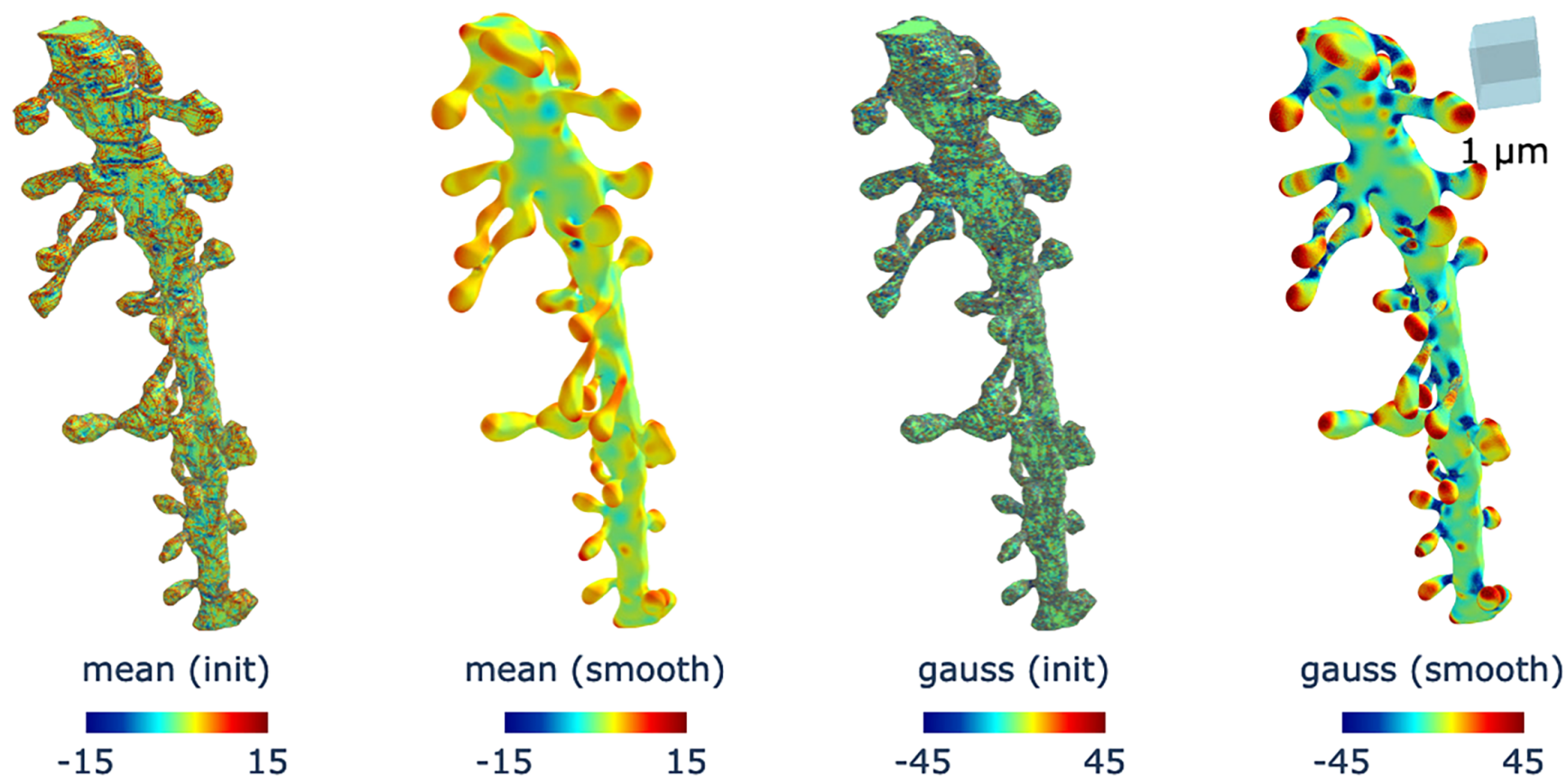

Figure 1. Effect of curvature smoothing on dendritic meshes. The plots labeled (init) and (smooth) show the initial dendritic curvature and the curvature after smoothing, respectively. The plots labeled mean and gauss correspond to the mean curvature and Gaussian curvature of a segment of dendrite. Smoothing enhances the curvature profile, producing a more easily interpretable pattern in the mesh that can be effectively leveraged to improve segmentation accuracy. For visualization, Gaussian curvature values are thresholded to remain within an absolute value of 45, while the mean curvature is constrained within an absolute value of 15, thereby highlighting the most relevant mesh faces. The triangular mesh is obtained from CA1 dendritic reconstructions.[28–30]

conditions, the curvature fields more accurately capture the cylindrical shape of the dendritic shaft and the geometric deviations introduced by spine necks and spine heads. As a result, the smoothed curvature values provide a more reliable representation of the underlying morphology, which is valuable in distinguishing the shaft from protruding spines.

Smoothing the triangulated surface mesh

To analyze the triangulated surface of the dendritic mesh, we first address the inherent roughness introduced by the EM sectioning process. We apply discrete differential-geometry techniques—specifically a discrete approximation of mean-curvature (Willmore) flow—to smooth the triangular surface mesh.

We define k_{bend} as the bending coefficient of the surface. The curvature energy over the surface A is

$$\mathbf{W}_{bend}(\mathbf{X}) = \frac{k_{bend}}{2}\int_A \mathbf{H}^2(\mathbf{X})\, dA, \tag{1}$$

and the associated bending force is obtained as the negative gradient of this energy:

$$\mathbf{F}_{bend}(\mathbf{X}) = -\nabla_{\mathbf{X}}\mathbf{W}_{bend}(\mathbf{X}). \tag{2}$$

The surface is then evolved by a gradient-descent step,

$$\frac{\partial}{\partial t}\mathbf{X} = \mathbf{F}_{bend}(\mathbf{X}), \tag{3}$$

which produces a smoothed version of the mesh. In practice, this smoothing is implemented using standard discrete differential-geometry operators. Although the mesh consists of discrete triangles, smoothing is well defined: each vertex exchanges curvature information only with the vertices in its 1-ring neighborhood (the set of triangles directly adjacent to it), as illustrated in Figure S1. Updating each vertex using curvature information from its neighbors provides a discrete analog of smoothing on a continuous surface. This suppresses high-frequency numerical noise introduced by the discretization while preserving the underlying geometric structure of the dendritic mesh. Further numerical details, following the approach of Wu et al.,[27] are provided in Section S5.

The results of performing this smoothing are illustrated in Figure 1, where we compute the Gaussian and mean curvature of a segment of spiny dendrite. The data were obtained from high-resolution 3D EM reconstructions of dendritic segments in the CA1 region of the hippocampus from P21 rats. Slices were subjected to *in vitro* theta-burst stimulation to induce long-term potentiation (LTP) or to control stimulation, as described elsewhere.[28–30] Comparing the curvature before and after smoothing, we observe that the processed mesh exhibits a more easily interpretable profile of the dendritic surface, which can be exploited to improve the accuracy of spine-shaft segmentation.

Enhancement of curvature through image processing

Here, we provide an intuition for using image-processing techniques to enhance the mean and Gaussian curvature values. The goal is to emphasize regions of the surface with significant curvature changes, which are more likely to correspond to dendritic shafts or spines, thereby facilitating segmentation. This forms the foundation for the machine-learning methods that will be developed in the segmentation algorithm.

First, let us define the following sigmoidal function, which is often used in image processing to normalize and enhance contrast:

$$\zeta(x) = \frac{1}{1+\exp(-x)}.$$

This transformation maps all real values into the interval (0, 1). For large positive x, $\zeta(x) \rightarrow 1$, while, for large negative x, $\zeta(x) \rightarrow 0$. Around $x = 0$, the function has its steepest slope, which enhances small variations near zero and makes them more distinguishable.

Applying this transformation to the mean curvature $\mathbf{H}$ and the Gaussian curvature $\mathbf{K}$, we obtain

$$\widetilde{\mathbf{H}} = \zeta(a_{\mathbf{H}}\mathbf{H} + b_{\mathbf{H}}), \quad \widetilde{\mathbf{K}} = \zeta(a_{\mathbf{K}}\mathbf{K} + b_{\mathbf{K}}), \qquad (4)$$

where $a_{\mathbf{H}}$, $b_{\mathbf{H}}$, $a_{\mathbf{K}}$, and $b_{\mathbf{K}}$ are empirically chosen parameters used to emphasize specific geometric features of the dendritic mesh. For example, in the neck region of a spine, we expect relatively high negative Gaussian curvature. By appropriately choosing $a_{\mathbf{K}}$ and $b_{\mathbf{K}}$, these negative values are pushed toward the lower end of the sigmoid, making them stand out more clearly during segmentation.

In contrast, along the cylindrical shaft, the Gaussian curvature is close to zero. Proper tuning ensures that values near zero are mapped consistently with the shaft so that these regions are correctly identified. For mean curvature, which distinguishes concave and convex regions, the transformation highlights transitions: concave regions (positive mean curvature) are enhanced toward higher sigmoid values, while convex regions (negative mean curvature) are mapped toward lower values.

In practice, within this paper, the quantities $\widetilde{\mathbf{H}}$ and $\widetilde{\mathbf{K}}$ are not computed explicitly. Their formulation serves only to motivate the use of a DNN as an approximation function for the segmentation task, with a sigmoidal activation used in the output layer. Figure 3 shows the spine and shaft probability outputs of the DNN that will be examined next. These results demonstrate a substantial improvement in segmentation quality compared to the manually tuned baseline. The overall processing pipeline—from mesh smoothing and geometric feature extraction to DNN-based prediction—is summarized in the flowchart shown in Figure S2.

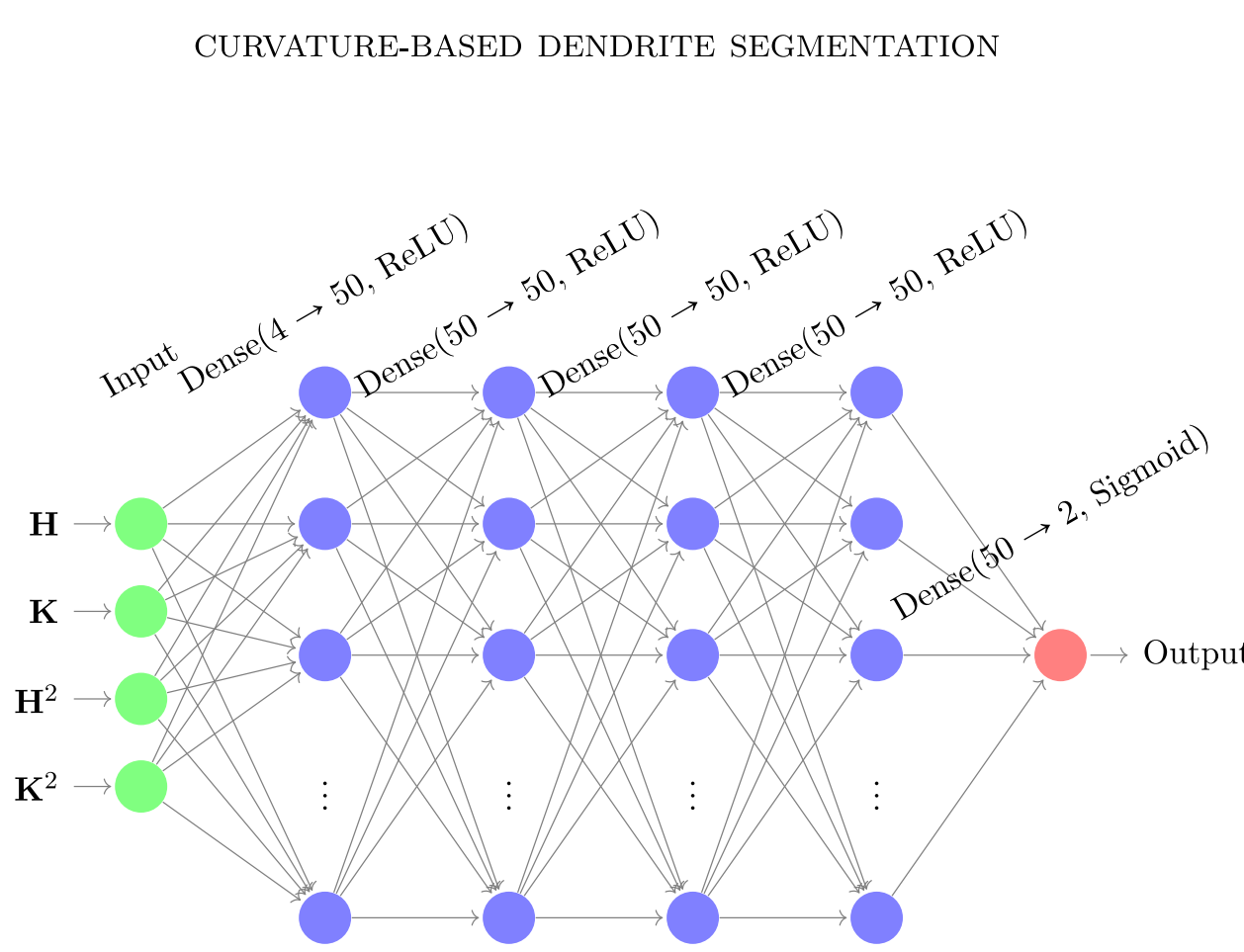

Figure 2. DNN_1 architecture used to segment the dendrite shaft. The input layer consists of the mean curvature $\mathbf{H}$ and the Gaussian curvature $\mathbf{K}$, computed after smoothing, and their squares. The network includes four hidden layers, each with 50 neurons and ReLU activation functions. The output layer employs a sigmoidal activation function.

DNN approach for spine and shaft analysis

In the previous section, we provided an intuitive explanation of how segmentation can be enhanced using image-processing techniques. We then introduced empirical filtering parameters that can improve segmentation quality. Instead of manually selecting these parameters and explicitly computing $\widetilde{\mathbf{H}}$ and $\widetilde{\mathbf{K}}$, we can leverage DNNs to learn an optimal segmentation approximation function based on $\mathbf{H}$ and $\mathbf{K}$. At the same time, DNNs allow us to incorporate non-linearities that further enhance segmentation performance.

We analyze three different DNN architectures. The first network is designed to support the second by assisting in the extraction of the shaft skeleton, while the third network relies solely on external Python libraries for skeletonization. Machine-learning methods have also been employed for dendrite segmentation of dendritic spines obtained from confocal reconstruction images using CNNs.[20] In this work, we adopt a simple DNN architecture inspired by the physics-informed neural-network (PINN) model,[31–38] which has been widely applied to approximate ordinary and partial differential equations. These models leverage their well-known ability to serve as universal function approximators.[37] Given the geometrical characteristics of the dendritic triangular mesh, this approximation capability of DNNs constitutes a fundamental tool in our algorithm. Here, we present the architectures that produced the best results among the different trials.

*DNN (**DNN$_1$**) using the Gaussian and mean curvature*

As for the sigmoidal function (4) introduced in the previous section, various model parameters require fine-tuning to enhance the shaft segmentation process. Rather than manually selecting these parameters and computing $\widetilde{\mathbf{H}}$ and $\widetilde{\mathbf{K}}$, we train a DNN in a two-step procedure.

We first train a DNN, denoted as DNN_1, whose architecture is illustrated in Figure 2. The input layer receives both the mean curvature $\mathbf{H}$ and the Gaussian curvature $\mathbf{K}$ values of the dendritic triangular mesh (after smoothing), together with their squared terms to capture higher-order variations. The input features are preprocessed such that the Gaussian curvature values and their squared terms are thresholded to remain within an absolute value of 45. Specifically, for $\mathbf{K} = (\mathbf{K}_1, \mathbf{K}_2, \ldots, \mathbf{K}_m)$, we enforce $\mathbf{K}_i \in [-45, 45]$. We then compute $\mathbf{K}_i^2$ and threshold its value to the range [0, 45]. Similarly, the mean-curvature values and their squared terms are constrained within an absolute value of 15, following the same procedure as in the Gaussian curvature case.

These thresholds are applied because curvature values can sometimes become very large, and such extreme values do not significantly improve prediction accuracy but can instead cause instability during training and inference. To further reduce instability, any NaN values, if present, are

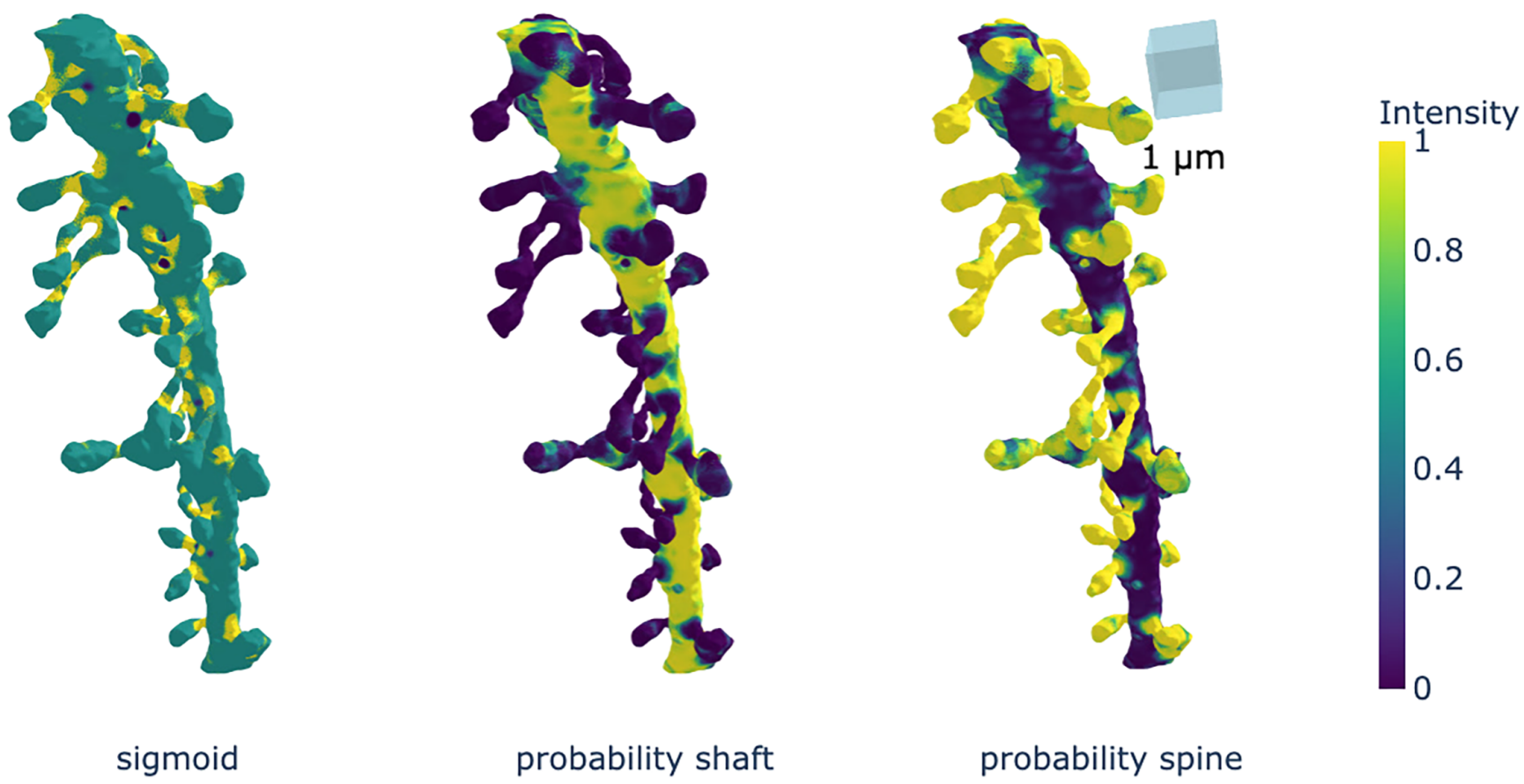

Figure 3. Visualization of geometric-based and learned probability representations used for dendritic spine segmentation. The part labeled “sigmoid” shows the sigmoid of a linear combination of mean intensity and Gaussian curvature, which highlights the neck region and serves as a handcrafted baseline cue for segmentation. The parts labeled “probability shaft” and “probability spine” display the class-specific probability outputs of the network DNN_3 for shaft and spine, respectively. Compared with the sigmoid representation, the learned probabilities provide a clearer and more discriminative separation of spine and shaft regions. The triangular mesh is obtained from CA1 dendritic reconstructions.[28–30]

replaced with the average of the corresponding curvature values.

The network consists of four hidden layers, each containing 50 neurons with Rectified Linear Unit (ReLU) activation functions to introduce non-linearity.[38] The output layer employs a sigmoid activation function, as described in section enhancement of curvature through image processing, to classify vertices into dendritic shafts and spines.

The segmentation produced by DNN_1 is not fully satisfactory, as regions within spines are sometimes misclassified as part of the shaft. This misclassification arises because certain spines regions exhibit relatively flat curvature, making them appear similar to shaft regions.

Skeletonization

In this section, we describe that the process used to obtain the skeletonization of dendritic meshes is an image-processing technique that reduces binary shapes to thin, single-pixel-wide lines while preserving their topological structure and connectivity.[39,40]

The first step in building the skeleton is to ensure that the mesh is watertight, meaning that it forms a completely closed surface with no gaps, holes, or disconnected edges. A watertight mesh guarantees a well-defined interior and exterior, which is essential for reliable geometric processing and for preserving the topological structure during skeletonization. To achieve this, we wrap the existing mesh using the algorithm described in Section S6.1. This procedure converts the surface into a uniformly sampled point cloud, estimates and orients normals, and then applies Poisson surface reconstruction to generate a closed, watertight representation.

In addition to Poisson reconstruction, we also evaluated the alpha-wrap method provided by `PyMeshLab`,[41] which is based on the classical α-shape formulation.[42] Alpha wrapping produces well-behaved closures and can preserve fine geometric detail; however, in our experiments, it was substantially slower than Poisson surface reconstruction for meshes of this size and resolution. For this reason, we adopted Poisson reconstruction as our primary wrapping method. For completeness, the alpha-wrap implementation is also included in Section S6.1.

Once a watertight mesh is obtained, we compute its skeleton using the `scikit-image` skeletonization package, which implements algorithms from Lee et al. and Zhang and Suen.[39,40] Further implementation details are provided in Section S6. This simplified representation captures the essential branching geometry of dendrites for subsequent segmentation and morphological analysis.

*DNN (**DNN$_2$**) using shaft skeleton*

We can enhance dendritic spine-shaft segmentation by incorporating the shaft skeleton. This involves using the distance between the shaft-skeleton vertices and the dendritic branch as an additional input to the DNN. For this purpose, we use the shaft segmented with DNN_1 together with the skeletonization procedure outlined in section skeletonization.

To begin, we consider the shaft-segmented meshes obtained from DNN_1. Since this mesh results from the dendritic branch after spine regions have been segmented, it is no longer watertight. Therefore, we apply the procedure described earlier to make the mesh watertight. Once the shaft mesh skeletonization is completed, we compute the distance $\mathbf{D}$ between the shaft skeleton and the dendritic-branch mesh vertices. Here, for each vertex $\mathbf{X}_l$, $l \in \{1, 2, \ldots, n\}$ in the dendritic triangular mesh with n vertices, and $\{\mathbf{V}_1, \mathbf{V}_2, \ldots, \mathbf{V}_v\}$ the set of shaft-skeleton vertices, we compute the Euclidean norm:

$$\mathbf{D}_l = \min_{1 \le j \le v} \left\| \mathbf{V}_j - \mathbf{X}_l \right\|_2, \qquad \mathbf{D} = (\mathbf{D}_1, \mathbf{D}_2, \ldots, \mathbf{D}_n). \tag{5}$$

With this additional information, we develop an improved DNN, DNN_2, whose architecture is shown in Figure S3. This model is similar to DNN_1 except that it incorporates the new input feature. As in the earlier model, the input layer

receives both the Gaussian and mean-curvature values of the dendrite, along with the squared values of these curvatures as additional features. The network consists of four hidden layers, each containing 50 neurons with ReLU activation functions to introduce non-linearity. The output layer employs a sigmoid activation function, as discussed in section enhancement of curvature through image processing, to classify vertices into dendritic shafts or spines.

*Dendrite spine-shaft segmentation using dendritic-branch regions (**DNN$_3$**)*

To improve segmentation accuracy, we introduce a DNN (DNN$_3$) that incorporates regional information from dendritic-branch segments. These segments are derived from the dendrite skeleton, which serves as a structural reference for spatial organization.

For this model, once the skeleton is extracted, we compute the shortest distance from each mesh vertex to its nearest skeleton point. These distances are then clustered using the *k*-means algorithm to partition the dendritic mesh into multiple regions. This regional segmentation enables us to distinguish between different parts of the dendrite shaft, spine neck, and spine head—regions that are critical for accurate classification.

As shown in Figure 4, to capture variations in dendritic morphology, we apply *k*-means clustering with multiple values of *k*. We denote these segmentation features by $\mathbf{S}^k$, where $k \in \{2, 3, \ldots, 10\}$ corresponds to the number of clusters. In particular, the value of each feature $\mathbf{S}^k$ is given by the corresponding cluster label. Therefore, this model incorporates nine additional scalar features. In this study, we explore values of k ranging from 2 to 10 to provide a multi-scale representation of regional structure.

In addition to these region-based segmentation features, we incorporate Gaussian curvature and its squared value as geometric descriptors. These measures are particularly effective for identifying neck regions, as discussed in earlier sections. We intentionally exclude mean curvature as we have observed that the mean-curvature input increases the probability of misclassifying all dome-like regions as spines, leading to false positives. By omitting mean curvature and focusing on more discriminative features, DNN$_3$ achieves improved segmentation performance compared to the baseline models.

Note that, in practice, the *k*-means algorithm is not deterministic because its initial values depend on a random seed. To mitigate this and obtain consistent region assignments, we perform 50 independent clustering runs and select the labeling corresponding to the run with the lowest final *k*-means inertia. Since inertia measures the within-cluster compactness optimized by *k*-means, choosing the run with minimal inertia yields the most stable and coherent clustering among all runs. This procedure effectively filters out seed-dependent fluctuations and provides reproducible region assignments without requiring *post hoc* consensus voting.

We also considered using the silhouette score as an alternative selection criterion; however, computing silhouette values for all vertices is prohibitively expensive due to the large number of mesh vertices. For this reason, inertia-based selection offered a practical and computationally efficient solution.

Loss function

We use the binary cross-entropy (BCE) to compute the loss function, and its derivation is presented below.

Let us consider a set of m dendritic meshes, denoted as $\mathcal{D} = (\mathcal{D}_1, \mathcal{D}_2, \ldots, \mathcal{D}_m)$, where each mesh $\mathcal{D}_i$ consists of n_i vertices. For the training set, we assume that each $\mathcal{D}_i$ has a ground-truth classification matrix $\mathbf{Y}_i \in \{0, 1\}^{n_i \times 2}$, which represents the classification of vertices in $\mathcal{D}_i$ in one-hot encoding, such that

$$\mathbf{Y}_{i,j} = \begin{cases} (0,1) & \text{if vertex } j \text{ of } \mathcal{D}_i \text{ belongs to the dendritic shaft,} \\ (1,0) & \text{otherwise.} \end{cases}$$

We define $\mathbf{Y} = (\mathbf{Y}_1, \mathbf{Y}_2, \ldots, \mathbf{Y}_m)$ as the set of all annotation matrices. Next, we define the set of mean-curvature vectors for all dendritic meshes as $\mathbf{H} = (\mathbf{H}_1, \mathbf{H}_2, \ldots, \mathbf{H}_m)$ and the set of Gaussian curvature vectors as $\mathbf{K} = (\mathbf{K}_1, \mathbf{K}_2, \ldots, \mathbf{K}_m)$. For

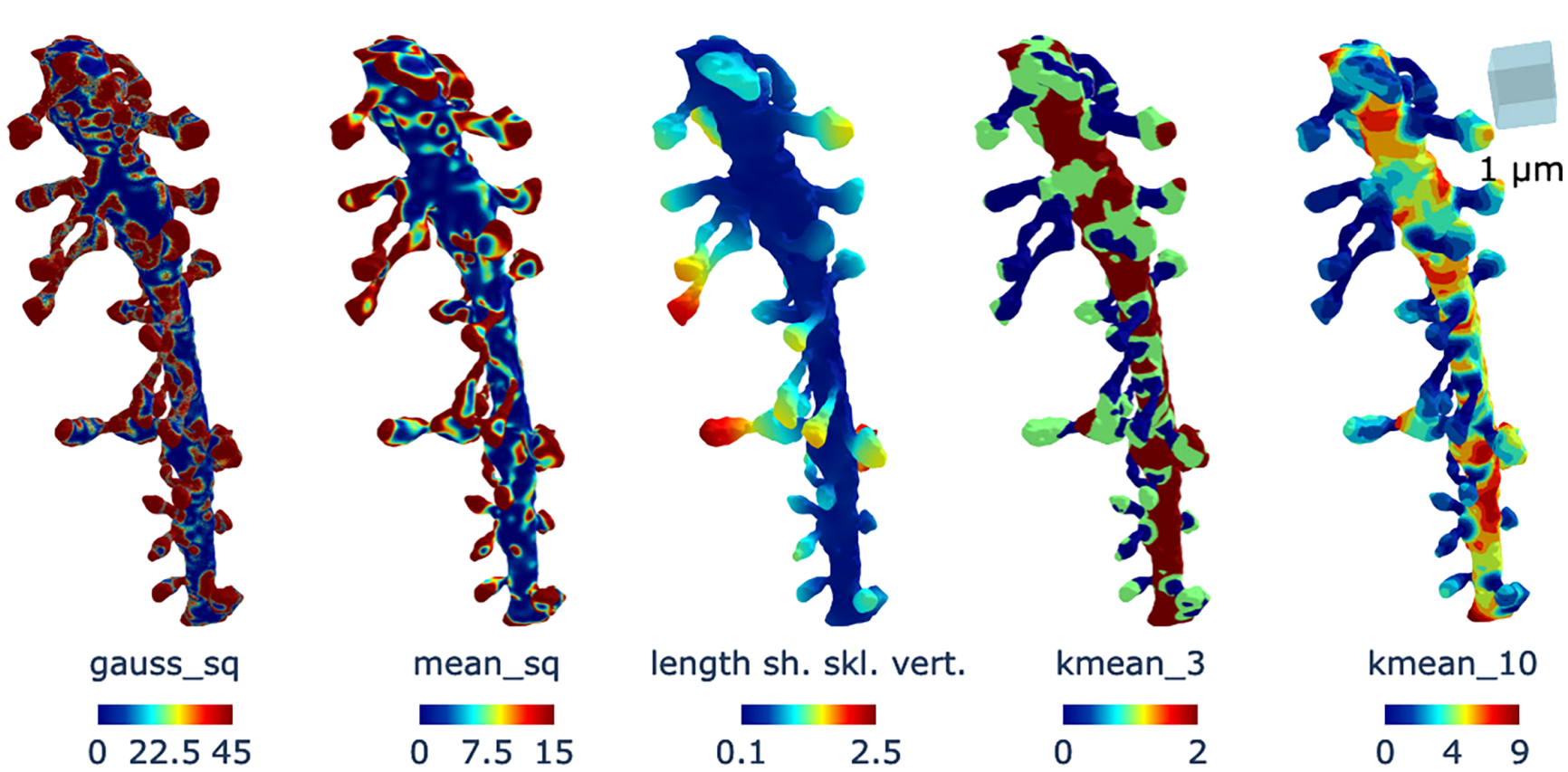

Figure 4. Additional features used in the DNN training. The plot labeled "gauss_sq" and "mean_sq" show, respectively, the square of the Gaussian and mean curvature of the dendrite. The plot labeled "length sh. skl. vert." indicates the distance from the shaft skeleton to the dendritic mesh vertices, denoted by **D**. The plot labeled "kmean_*k*" illustrates the *k* subdivision from *k*-means clustering, corresponding to the set $\mathbf{S}^k$. The triangular mesh is obtained from CA1 dendritic reconstructions.[28–30]

each mesh $\mathcal{D}_j, j \in \{1, 2, ..., m\}$, the mean-curvature vector is given by $\mathbf{H}_j = (\mathbf{H}_{1,j}, \mathbf{H}_{2,j}, \ldots, \mathbf{H}_{n_j,j})$, and the Gaussian curvature vector by $\mathbf{K}_j = (\mathbf{K}_{1,j}, \mathbf{K}_{2,j}, \ldots, \mathbf{K}_{n_j,j})$, where $\mathbf{H}_{i,j}$ and $\mathbf{K}_{i,j}$ denote the mean and Gaussian curvatures of $\mathcal{D}_j$ at vertex i, for $i \in \{1, 2, ..., n_j\}$.

Additionally, we define the distance vector $\mathbf{D} = (\mathbf{D}_1, \mathbf{D}_2, \ldots, \mathbf{D}_m)$, representing the distances between the central curve and the vertices, as well as additional structural descriptors $\mathbf{S}^k$, where $k \in \{2, 3, \ldots, 10\}$. For each mesh $\mathcal{D}_j$, we write $\mathbf{S}_j^k = (\mathbf{S}_{1,j}^k, \mathbf{S}_{2,j}^k, \ldots, \mathbf{S}_{n_j,j}^k)$.

We now define the feature vector for vertex i of mesh j as

$$\mathbf{Z}_{i,j} = \left(\mathbf{H}_{i,j}, \mathbf{H}_{i,j}^2, \mathbf{K}_{i,j}, \mathbf{K}_{i,j}^2, \mathbf{D}_{i,j}, \mathbf{S}_{i,j}^2, \mathbf{S}_{i,j}^3, \ldots\right),$$

and the corresponding feature matrix for dendritic mesh $\mathcal{D}_j$ as

$$\mathbf{Z}_j = \left(\mathbf{H}_j, \mathbf{H}_j^2, \mathbf{K}_j, \mathbf{K}_j^2, \mathbf{D}_j, \mathbf{S}_j^2, \mathbf{S}_j^3, \ldots\right).$$

Our objective is to find a function

$$f_\theta : \mathbf{Z}_j \mapsto \mathbf{Y}_j \in [0,1]^{n_j \times 2}, \quad \forall j \in \{1, 2, \ldots, m\},$$

that best approximates the ground-truth classification by minimizing the following loss function:

$$\mathcal{L} = \min_\theta \|f_\theta(\mathbf{Z}) - \mathbf{Y}\|,$$

where $\|\cdot\|$ denotes an appropriate norm measuring the discrepancy between the predicted and actual classifications. In particular, we use a weighted sum of the BCE, which encourages a more balanced optimization process. The BCE formula is given by

$$\mathrm{BCE}(y, \hat{y}) = -(y \log(\hat{y}) + (1-y)\log(1-\hat{y})).$$

The final loss function is defined as

$$\mathcal{L}_\theta = \frac{1}{m}\sum_{j=1}^{m}\left(\frac{1}{n_j}\sum_{i=1}^{n_j}\left(\mathrm{BCE}(\mathbf{Y}_{i,j}, f_\theta(\mathbf{Z}_{i,j}))\cdot w_j\right)^2\right).$$

Here the weights w_j is empirically determined.

Note that the mean curvature $\mathbf{H}$ and Gaussian curvature $\mathbf{K}$ used during training are computed after smoothing the curvature fields on the triangular dendritic-branch meshes. The sole purpose of this smoothing is to obtain stable and interpretable curvature estimates that enhance the segmentation performance during training. As described in section smoothing the triangulated surface mesh, smoothing the curvature improves the readability of the curvature profile and reduces noise-driven artifacts. Thus, the smoothing step in our pipeline serves only as a feature-enhancement operation, not as a geometric modification of the underlying dendrite.

Importantly, all manual annotations were performed on the actual, unsmoothed dendritic branches, ensuring that the ground-truth labels correspond to the true biological geometry. During training, the network is therefore supervised to segment the real dendritic shaft and spine structures. After training, all dimensional measurements and quantitative analyses are performed on the original triangulated meshes, not on the smoothed versions.

Spine and shaft detection

After the training step of the algorithm, the next stage of spine-shaft segmentation involves applying the model for classification as well as performing additional post-processing steps to first isolate the shaft and then the spines. In this section, we analyze the processes required to obtain a reliable segmentation.

Grouping dendritic mesh parts into connected vertices

After training the DNN, the first step is to classify the vertices of a given test dendritic mesh into shaft and spine categories. The resulting classification can then be grouped into connected components of spine vertices and shaft vertices.

As a consequence of the sigmoidal activation function applied to the final layer, the output of the DNN is a probability matrix $\overline{\mathbf{Y}} = f_\theta(\mathbf{Z}) \in [0,1]^{n \times 2}$, where each row corresponds to a vertex and contains the probabilities of that vertex being classified as a spine, $\widetilde{\mathcal{X}}_{\text{spine}}$ (first column greater than the second), or a shaft, $\widetilde{\mathcal{X}}_{\text{shaft}}$ (second column greater than or equal to the first). Formally, we define

$$\begin{aligned}\widetilde{\mathcal{X}}_{\text{spine}} &= \{\mathbf{X}_i \epsilon \mathcal{D} | b\overline{\mathbf{Y}}(i,1) > a\overline{\mathbf{Y}}(i,0)\},\\ \widetilde{\mathcal{X}}_{\text{shaft}} &= \{\mathbf{X}_i \epsilon \mathcal{D} | b\overline{\mathbf{Y}}(i,1) \leq a\overline{\mathbf{Y}}(i,0)\}.\end{aligned} \tag{6}$$

Here, a and b are user-defined weighting parameters that control the decision boundary. In our experiments, we fixed $b = 0.81$ and initially set $a = 3$. If the resulting segmentation produced too many shaft labels, we increased a; if too many spine labels were produced, we decreased a. This adjustment allowed us to fine-tune the spine-shaft separation based on the model's output distribution.

Next, we describe how to group the predicted dendritic parts—classified as either spine or shaft —into subgroups of connected components. Let each vertex $\mathbf{X}_i$ have an associated set of neighboring vertices denoted by $\mathcal{N}_i$:

$$\mathcal{N}_i = \left\{\mathbf{X}_j \mid \mathbf{X}_j \text{ is in the } 1-\text{ring neighborhood of } \mathbf{X}_i\right\}.$$

We define a connected group of vertices $\mathcal{G}_i \subseteq \mathcal{D}$ such that, for any pair $\mathbf{X}_p, \mathbf{X}_q \in \mathcal{G}_i$, there exists a path of vertices $\mathbf{X}_{i_1}, \mathbf{X}_{i_2}, \ldots, \mathbf{X}_{i_m} \in \widetilde{\mathcal{X}}_{\text{part}}$ satisfying

$$\mathbf{X}_{i_1} = \mathbf{X}_p, \quad \mathbf{X}_{i_m} = \mathbf{X}_q, \quad \mathbf{X}_{i_\ell} \in \mathcal{N}_{i_{\ell-1}} \quad \text{for all } \ell = 2, \ldots, m.$$

Here, $\widetilde{\mathcal{X}}_{\text{part}}$ denotes the set of vertices classified as a given part type; either $\widetilde{\mathcal{X}}_{\text{spine}}$ for spines or $\widetilde{\mathcal{X}}_{\text{shaft}}$ for shafts. The subset of part-classified vertices within this group is then

$$\widetilde{\mathcal{Y}}_{\text{part}}^i = \left\{\mathbf{X}_j \in \widetilde{\mathcal{X}}_{\text{part}} \mid \mathbf{X}_j \in \mathcal{G}_i\right\}.$$

Finally, the complete set of part-classified vertices $\widetilde{\mathcal{Y}}_{\text{part}}$ can be decomposed into disjoint set of connected vertices, where connectivity is defined by neighborhood overlap:

$$\tilde{\mathcal{Y}}_{\text{part}} = \left\{ \tilde{\mathcal{Y}}^i_{\text{part}} \right\}_i.$$

Spine-shaft detection

Using the subgroups defined above of connected components, we now define the segmentation of the dendritic mesh into spines and shaft. Let $\widetilde{\mathcal{Y}}_{\text{shaft}}$ denote the set of connected components classified as shaft. In practice, some of these components may actually be parts of spines, so it is crucial to separate them from the true shaft set for accurate segmentation.

To achieve this, we identify the largest connected component in $\widetilde{\mathcal{Y}}_{\text{shaft}}$ and designate it as the entire shaft. All remaining connected components in $\widetilde{\mathcal{Y}}_{\text{shaft}}$ are then assumed to belong to spines.

The set of vertices belonging to the shaft is defined as

$$\mathcal{Y}^0_{\text{shaft}} = \underset{\widetilde{\mathcal{Y}}^i_{\text{shaft}} \subset \widetilde{\mathcal{X}}_{\text{shaft}}}{\operatorname{argmax}} \left| \widetilde{\mathcal{Y}}^i_{\text{shaft}} \right|,$$

where $|\cdot|$ denotes the cardinality of the set.

Once the entire shaft is defined, spine segmentation proceeds by removing the shaft vertices from the dendrite vertex set, reclassifying them as spine vertices, and then reapplying the connected-component grouping process described in section grouping dendritic mesh parts into connected vertices to the remaining vertices. The set of segmented spines is defined as

$$\mathcal{Y}_{\text{spine}} = \left\{ \mathcal{Y}^i_{\text{spine}} \right\}_i,$$

where each individual spine component is given by

$$\mathcal{Y}^i_{\text{spine}} = \left\{ \mathbf{X}_j \in \mathcal{D} \backslash \mathcal{Y}^0_{\text{shaft}} \mid \mathbf{X}_j \in \mathcal{G}_i \right\}.$$

Dataset description

This section describes the datasets of dendritic segments used for training and testing our algorithm. To mitigate overfitting, the training and testing sets were independent and originate from different animals. This separation ensures that the model learns generalizable features of dendritic structures rather than memorizing patterns specific to a single specimen. Table S3 provides a consolidated overview of the datasets used for training and testing, including their sources, annotation strategy, and selection criteria.

Training dataset

The training dataset used as ground truth in this paper comprises six high-resolution 3D EM reconstructions of dendrites from the CA1 region of the hippocampus from rats at postnatal day 21 (P21), an immature developmental stage. Brain slices from these animals either underwent *in vitro* theta-burst stimulation to induce LTP or received control stimulation, as described elsewhere.[28–30]

For the training dataset, the meshes were manually annotated by experts, where spine and shaft segmentations were performed on all six dendritic-branch reconstructions. Each spine was stored as an individual `.obj` file in watertight format. The mesh corresponding to the shaft of each dendritic segment was provided separately, along with the complete mesh of the dendritic segment. Because spine and shaft meshes were stored independently, we wrapped the spines and shaft with a tight mesh from the exterior in order to generate a unified dendritic mesh using the algorithm in Section S6.1. We then used the independent spine meshes to label the constituent parts of the new wrapped dendritic mesh. To achieve this, we employed a KD-tree-based nearest-neighbor search to map spine vertices to the wrapped dendritic mesh. Specifically, for each set of spine vertices in the individual dataset `vertices`, we queried a KD tree built from the wrapped dendritic mesh vertices to identify all branch vertices within a radius threshold r_{th}:

$$\texttt{vertices\_appr} = \texttt{list(set(np.concatenate}$$
$$\texttt{(kdtree.query\_radius(vertices,}\, r_{\text{th}}\texttt{)))).}$$

Here, `query_radius` returns the indices of dendrite mesh vertices that lie within a distance r_{th} of each spine vertex. By aggregating these indices, we obtain the set of dendrite vertices corresponding to the annotated spine regions on the wrapped mesh. This mapping step allows us to merge the spine and shaft meshes into a single labeled dendritic mesh, which is then used for both training and validation.

Training data augmentation

Only six dendritic branches are available for training, so we expanded the dataset by applying a series of geometric transformations. First, we computed the principal-component analysis (PCA) of each mesh and projected its vertices onto the corresponding PCA plane. We then applied random scaling to the vertices in two separate steps, introduced random rotations in two additional steps, and added random translations. These transformations produced four augmented versions of each original mesh, resulting in a total of 30 dendritic branches available for training and validation. Of these, 22 branches were used for training, and the remaining eight were used for validation.

Testing dataset

For testing, we used data from the axon-spine coupling study, which includes a complete nanoconnectomic 3D reconstruction of hippocampal neuropil obtained via serial EM.[43] This dataset contains four independent annotations—two performed by each of two annotators[44]—providing detailed segmentation of dendritic spines. Out of 151 meshes, we selected 28 spiny dendritic branches

for testing based on annotation consensus. Specifically, we included only those meshes where two or more annotators agreed on the presence of at least one dendritic spine. Additionally, for each spine mesh identified by the annotators, we retained only those meshes that were consistently labeled as spines by at least two of the selected annotations. This ensured that the testing set reflected a high-confidence subset of spiny dendritic structures.

It is important to note that some structures exhibiting spine-like morphology were not annotated as spines in the test set for two reasons. First, biologically, the presence of a synaptic area in the spine head is essential for defining a spine. Thus, even if a mesh appears spiny, it is not considered a spine if no synapse is present. Second, some meshes were excluded due to incomplete reconstruction—parts of the spine may have been cut off or lost during imaging.

In contrast, the training dataset does not apply as strict criteria for spine identification as the test set. As a result, the testing dataset introduces additional segmentation challenges that are not reflected in the training data. Our algorithm does not explicitly account for these differences, which may influence performance.

Independent dataset

In addition to the training and testing datasets, we also used dendritic segments from the large-scale nanoconnectomic EM reconstruction of mouse neocortex by Kasthuri et al.[1] This dataset provides densely reconstructed neuropil at nanometer resolution but does not include manual spine or shaft annotations. We therefore use it solely for qualitative visualization (Figure 6) to demonstrate that our method generalizes to independently acquired EM volumes. These data are not used for training, validation, or quantitative evaluation.

RESULTS

This section presents a comprehensive evaluation of our algorithm's performance in segmenting dendritic shafts and spines. We first describe the identification of dendritic shafts using geometric properties and a neural-network-based approach. We then assess the ability of the algorithm to segment dendritic spines, highlighting both its strengths and limitations.

Figure 6 illustrates the predicted dendritic shafts and spines from datasets from Kasthuri et al.,[1] demonstrating that our method successfully identifies spines along the dendritic shaft by leveraging geometric properties such as Gaussian and mean curvature. However, misclassification can occur when the dendritic structure deviates significantly from the idealized cylindrical shape, underscoring the need for further improvements to the method.

DNN prediction results analysis

In this section, we present and analyze the prediction results of the three DNNs, DNN_1, DNN_2, and DNN_3. The methodology is outlined in section dendrite spine-shaft segmentation using dendritic-branch regions (DNN_3), and the evaluation here focuses on four key metrics: the training loss, the dice similarity coefficient (DICE), the Jaccard index intersection over union (IoU), and the area under the receiver operating characteristic curve (AUC).

Analysis of the performance

The training loss curves for the three models are shown in Figure S4. Among the three models, DNN_2 achieves the most effective convergence, reaching a minimum loss of approximately 2.44, closely followed by DNN_3 with a minimum loss of about 3.56. In contrast, the baseline architecture DNN_1 shows the weakest convergence, with a substantially higher final loss of 6.73 throughout training. These results highlight the importance of the additional features and architectural refinements incorporated into DNN_2 and DNN_3 for improving optimization stability.

To quantitatively assess segmentation accuracy, we computed the IoU, DICE, and AUC scores between predicted and annotated vertices for the dendritic shaft and spines on both the training and test sets. The performance curves are presented in Figure S5. For DNN_2, the IoU converges to average values of approximately 0.70 for the shaft and 0.81 for the spines, with corresponding DICE scores of about 0.88 and 0.92, respectively. DNN_3 achieves comparable performance across both IoU and DICE metrics, whereas DNN_1 lags behind with average IoU values of 0.55 (shaft) and 0.75 (spines), along with lower DICE scores overall. The AUC curves further support these trends: both DNN_2 and DNN_3 maintain consistently high AUC values throughout training, while DNN_1 again shows weaker discriminative performance. Importantly, the similarity between training and testing curves across all metrics indicates good generalization and minimal overfitting.

Overall, these results demonstrate a clear progression in performance from DNN_1 to DNN_3. The incorporation of additional geometric and topological features in DNN_2 and DNN_3 substantially improves convergence and segmentation accuracy, underscoring the value of feature enrichment in dendritic spine detection.

Dendritic spine detection analysis

Following dendritic shaft segmentation, we applied the spine detection algorithm described in section spine-shaft detection to identify spines. For each detected spine, we computed the IoU to evaluate segmentation accuracy. In addition, we calculated the IoU for the union of all detected spines to assess overall performance. We then computed

accuracy, precision, recall, and F1 score to further quantify classification performance.

The segmentation results produced by the DNN models are shown in Figure 5. A clear qualitative progression is evident from DNN_1 to the more advanced architectures DNN_2 and DNN_3. The DNN_1 model frequently misclassifies portions of the shaft as spines, resulting in fragmented and noisy predictions. Incorporating additional geometric features—such as the distance to the shaft skeleton—allows DNN_2 to reduce these errors and produce a more coherent separation between shaft and spine regions. Building on this, DNN_3 integrates richer geometric and topological descriptors, enabling it to capture subtle curvature variations and resolve complex spine clusters more effectively. These improvements are visible not only in the 3D mesh renderings but also in the 2D slice, where the highlighted region clearly shows the reduction in mislabeling from DNN_1 to DNN_3. Overall, the predicted segmentations from DNN_3 align most closely with the expert annotation. These qualitative trends are consistent with the quantitative results in Table S4, where both DNN_2 and DNN_3 outperform DNN_1 across all evaluation metrics.

Quantitative evaluation and analysis

We evaluated segmentation performance using multiple complementary metrics, including AUC, precision, recall, and F1 score, computed under both IoU and DICE criteria, each with standard and union variants. These results, summarized in Table S4, enable a detailed comparison between our geometric-informed models (DNN_1, DNN_2, DNN_3) and established CNN baselines (U-Net, VoxNet, VGG16-FCN). Details on the training procedures and evaluation of the three CNN models are provided in Section S9. The weighting parameters used in the geometric-informed models are specified in Equation 6, where the values of a for each model are listed and the parameter b is fixed at 0.81.

Among the CNN baselines, U-Net achieves the highest AUC (0.792), indicating strong global discriminative ability. However, AUC alone does not fully capture fine-scale segmentation quality. When examining precision, recall, and F1 score under the IoU and DICE criteria, the geometric-informed model DNN_3 consistently delivers the strongest performance. Under the IoU-union metric, DNN_3 attains the highest recall (0.873) and F1 score (0.845), indicating that it captures spine regions more completely than both the CNN baselines and the other geometric-informed variants. Its DICE-union performance is even stronger, with an F1 score of 0.857 and a recall of 0.895.

In contrast, DNN_1 and DNN_2 exhibit more limited recall, particularly under the standard variants of IoU and DICE, where both models tend to miss a substantial fraction of true spines despite maintaining moderate to high precision. VoxNet and VGG16-FCN perform noticeably worse across all metrics, with lower AUC values (0.682 and 0.466, respectively) and substantially reduced F1 scores, especially under the IoU criterion. These results highlight the limitations of intensity-based CNN architectures when applied to thin, high-curvature structures such as dendritic spines.

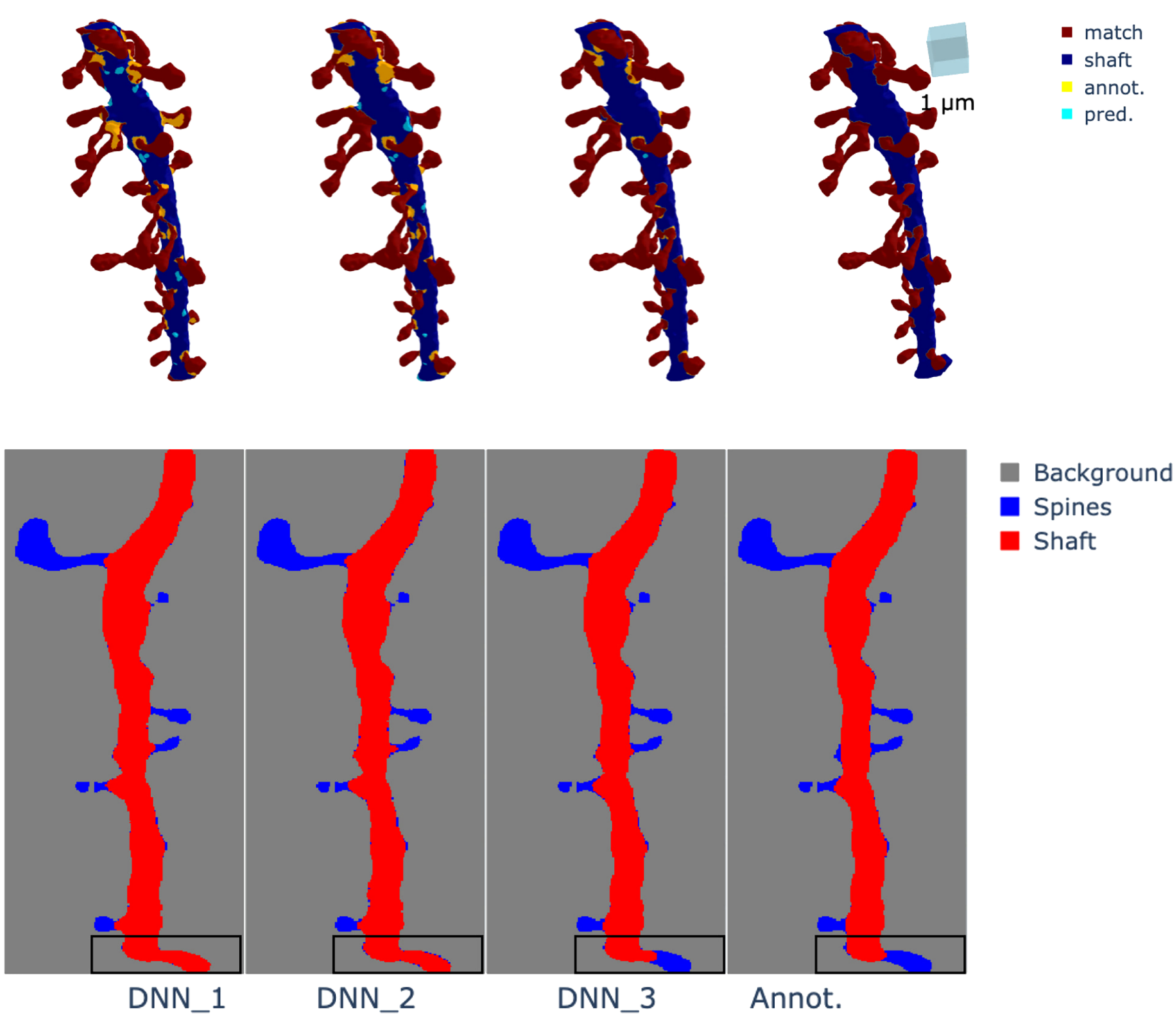

Figure 5. Comparison of segmentation results obtained using DNN_1, DNN_2, and DNN_3. The first three columns correspond to the predictions from DNN_1, DNN_2, and DNN_3, respectively, and the final column shows the expert spine annotation used as ground truth. The top row displays the 3D mesh representations, while the bottom row shows 2D slices of the same regions. In the meshes, red vertices indicate correct spine predictions that match the annotation, sky-blue vertices represent shaft regions misclassified as spines, and yellow vertices represent spine regions misclassified as shaft. DNN_1 produces noisier results with more misclassifications, particularly in clustered regions. DNN_2 reduces shaft-to-spine errors, and DNN_3 achieves the most accurate segmentation overall, with fewer misclassifications and the closest agreement with the expert annotation. The slice highlights the region where mislabeling is most apparent, especially for DNN_1 and DNN_2. The triangular mesh is obtained from CA1 dendritic reconstructions.[28–30]

The union variants of IoU and DICE consistently yield higher recall and F1 scores across all models, reflecting their ability to more fairly evaluate clustered or contiguous spine regions. This is particularly relevant in dense dendritic environments, where individual spine boundaries may be ambiguous. Under these more biologically meaningful metrics, DNN_3 shows the clearest advantage, outperforming all baselines by a substantial margin.

Overall, the quantitative results demonstrate a clear progression in performance from DNN_1 to DNN_3, with DNN_3 providing the most reliable and complete segmentation of dendritic spines. The strong performance of DNN_3 under both IoU and DICE—especially in the union variants—underscores the value of incorporating geometric curvature cues for accurate spine-shaft segmentation.

Dendrite spine segmentation without smoothing

In the segmentation of dendrite triangular meshes, curvature smoothing is typically required. However, when the number of vertices is large—more than 200,000—the process becomes computationally expensive, taking more than 20 h to produce a smooth mesh. We note that, by using DNN_3, the computational time can be reduced by performing segmentation without smoothing while still achieving a level of accuracy comparable to that obtained with smoothing.

We evaluated the effectiveness of curvature smoothing with DNN_3 on both the training and test datasets and compared the accuracy. The computed accuracy for the test dataset is 0.658 (with and without smoothing), while, for the training dataset, it is 0.704 with smoothing and 0.780 without smoothing. These results show that smoothing the curvature of the triangular mesh when using DNN_3 is unnecessary, and segmentation may even perform better without it.

This phenomenon may be due to the fact that the nine region-based segmentation features $\mathbf{S}^k$ enable the DNN to detect the neck area without requiring an enhanced curvature profile produced by smoothing. When we tested the same process on other DNN models, the accuracy was zero in both cases.

Application to dendritic surface meshes with many vertices

We next use our algorithm to segment the spines of a dendritic mesh dataset containing a large number of vertices and multiple branches.

In particular, the dataset we study is from the reconstruction of a sub-volume of mouse neocortex.[1] We analyzed a dendritic mesh with 5,387,879 vertices and 10,777,005 faces. This dendritic mesh is about 13.42 times larger than the training dataset used in the previous sections (401,371 vertices and 802,750 faces). While most of the training and testing datasets contain only one main dendritic branch with attached spines, this dataset has nine branches with multiple spines. This highlights how much more complex these dataset is compared to the training and testing data.

Because this dataset is relatively large, the segmentation process differs slightly from that used in training. Computations were performed on a MacBook Pro with an Apple M4 Max chip and 64 GB of memory. When we attempted to segment the large dendritic mesh directly, the process failed due to computational constraints. Therefore, we reduced the size of the mesh using the simplification algorithm described in Section S6.2. The mesh was simplified to nearly the size of the largest training dataset, given (about 449,109 vertices and 900,000 faces), and we then proceeded with the same testing process as before.

The segmentation results are shown in Figure 6. Overall, DNN_3 achieves the strongest performance, correctly labeling the majority of spines across the dendritic mesh. However, it still misclassifies a small region of the shaft as spines, as illustrated in Figure 6C. This error likely arises from local geometric similarities between the shaft surface and spine neck regions, particularly where the mesh bends or narrows.

In contrast, DNN_1 also performs reasonably well and produces few large false-positive spine regions. However, closer inspection of Figure 6A shows that DNN_1 frequently misclassifies spine necks as shaft, and in some cases entire spines are labeled as shaft. These errors are consistent with the limited feature set used by DNN_1, where Gaussian and mean curvature alone do not provide sufficient spatial context for reliable spine-shaft discrimination.

Finally, DNN_2 exhibits the weakest performance on this dataset. It correctly identifies spines in only a subset of regions while misclassifying large contiguous portions of the spines as shaft. This behavior suggests that the combination of shaft-skeleton distance, Gaussian curvature and mean-curvature features are insufficient to capture the complex local geometry present in this large-scale reconstruction.

Dendritic spine morphologic parameters

In this section, we use the segmentation results obtained from our algorithm to compute several morphological parameters of the dendritic meshes in both the training and testing datasets. These parameters include the neck diameter, head diameter, and spine length.

The computation of head and neck diameters was performed using the spine skeleton. This skeleton was obtained by identifying the closest point of the triangular mesh skeleton to each vertex of the segmented spine mesh. To determine the neck and head diameters, we considered each segmented spine's triangular mesh. For each vertex, we computed the shortest distance to its nearest skeleton point, as described in Equation 5, while computing $\mathbf{D}$. This procedure yielded a thickness profile along the spine. The

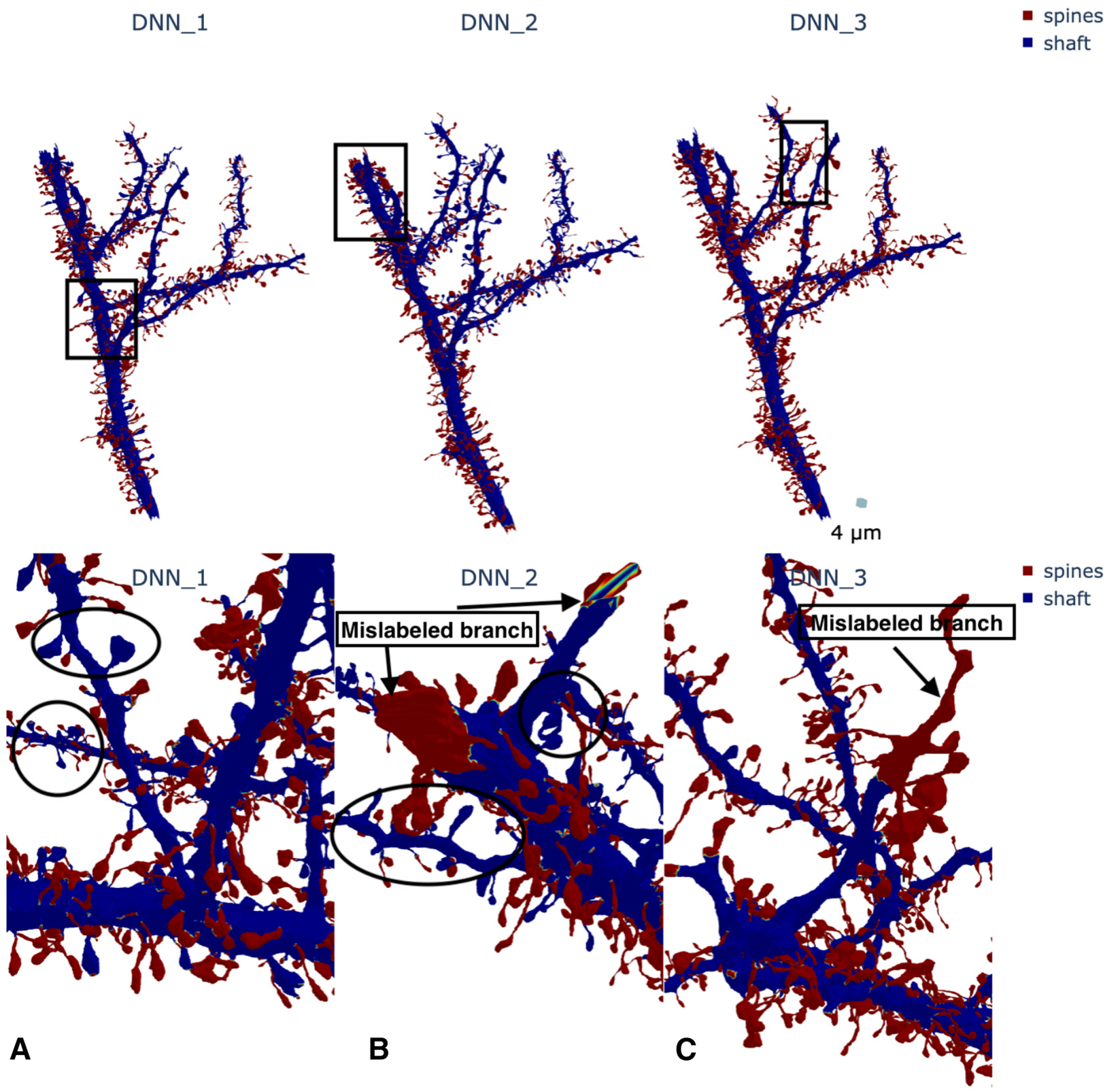

Figure 6. Comparison of segmentation results obtained using DNN_1, DNN_2, and DNN_3 on a large-scale nanoconnectomic EM reconstruction of mouse neocortex. In all parts, blue indicates the dendritic shaft mesh, while red highlights the segmented spines. The top part shows the full dendritic segment with predictions from the three models. All models correctly identify the majority of spines, but notable differences emerge upon closer inspection. Three boxed regions highlight areas of disagreement, which are shown in detail in the bottom parts. (A) Spine meshes that DNN_1 incorrectly labels as shaft. (B) Dendritic sub-branches that DNN_2 misclassifies as spines. (C) A similar misclassification pattern for DNN_3, where local curvature and branching geometry lead to spurious spine predictions. Reconstruction taken from Kasthuri et al.[1]

neck radius was then defined as the minimum of these computed averages, starting from the skeleton vertices closest to the dendritic shaft. Conversely, the head diameter was defined as the maximum computed distance, measured from the skeleton vertices at the farthest point from the shaft.

Additionally, to compute the length of the spine, we used the spine skeletonization and interpolated additional points along the skeleton using a spline technique. This interpolation increases the accuracy of the computed distances by smoothing and interpolating along the dendritic spine skeleton.[45–47] Additional details are provided in Section S7. The spine length was then computed as the sum of Euclidean distances between consecutive interpolated points:

$$L = \sum_{i=1}^{N-1} \|\mathbf{V}_{i+1} - \mathbf{V}_i\|_2,$$

where $\mathbf{V}_i$ denotes the i-th interpolated vertex.

The distribution of morphological parameters in the training and large dendritic mesh datasets is shown in Figure 7. In the first column, the head diameter is plotted against the neck diameter of dendritic spines, while in the second column the spine volume is plotted against the spine area, with a colormap encoding spine length. For clearer visualization, some outliers were removed. Marginal histograms of head and neck diameters are also included to facilitate interpretation.

Quantitatively, the segmented training dataset contains 210 spines, with an average neck diameter of 0.166 ± 0.0969 μm, an average head diameter of 0.512 ± 0.101 μm, and an average spine length of 1.35 ± 0.694 μm. The average spine volume is 0.122 ± 0.121 μm^3 and the average spine area is 2.30 ± 1.83 μm^2, yielding an average area-to-volume ratio of 12.9. Importantly, the relationship between spine volume and area is very strong, as reflected by a high coefficient of determination ($R^2 = 0.916$).

In contrast, the segmented large dendritic mesh dataset contains 562 spines, with an average neck diameter of 0.148 ± 0.0787 μm, an average head diameter of 0.661 ± 0.164 μm, and an average spine length of 5.57 ± 5.34 μm. The average spine volume is 0.090 ± 0.085 μm^3 and the average spine area is 1.53 ± 1.15 μm^2, yielding an average area-to-volume ratio of 13.9. Here too, the relationship between spine volume and area remains strong, with a coefficient of determination of $R^2 = 0.847$.

DISCUSSION

In this work, we developed and evaluated three geometric-informed DNN architectures for dendritic shaft and spine

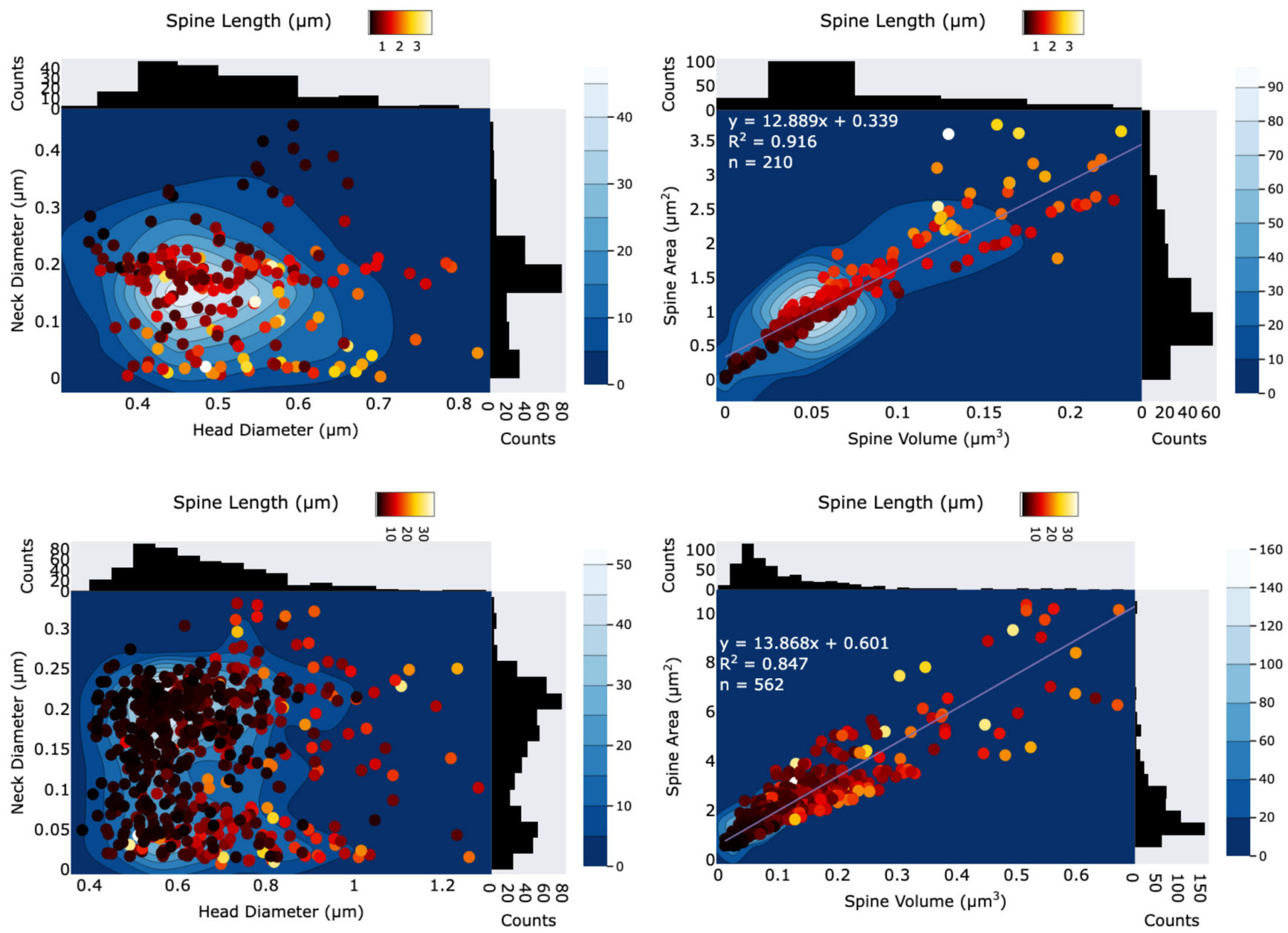

Figure 7. Distributions of morphological properties. Properties obtained using **DNN**$_3$ from the training dataset[44] (top row) and the large dendritic mesh dataset[1] (bottom row). In the first column, spine head diameter is plotted against spine neck diameter, while, in the second column, spine volume is plotted against spine area. The colormap encodes spine length.

segmentation. Our results demonstrate a clear progression in performance from the baseline DNN_1 to the enhanced models DNN_2 and DNN_3, with DNN_3 achieving the strongest overall segmentation accuracy. By incorporating curvature-based and topology-aware features, these models substantially improve both training stability and predictive performance, particularly in regions with dense spine clusters or complex branching patterns.

A key advantage of the geometric-informed approach is its computational efficiency relative to volumetric 3D CNNs. CNN-based models require dense voxel representations that scale cubically with resolution, making them memory intensive and slow when applied to high-resolution EM reconstructions. In contrast, our geometric models operate directly on mesh-derived features, avoiding voxelization and enabling faster inference on large or irregular geometries. As a result, DNN_3 not only surpasses all CNN baselines in accuracy but also scales more effectively to high-resolution meshes, making it the preferred choice for detailed EM datasets where preserving fine structural detail is essential.

The resulting distributions of spine size and shape connect directly to the fundamental biological question of how form determines function in dendritic spines.[3,26] In previous work,[48,49] we identified the neck radius as a key parameter controlling endocytic membrane trafficking and dendritic spine maintenance. The distributions generated here suggest that there are roughly bimodal distributions of spine neck diameters, which we conjecture may correspond to distinct physiological states in which vesicle entry is up- or downregulated. Although spine area and volume are observed to be positively correlated, as are the spine length and head diameter, we found only weak correlations between neck diameter and the other morphological properties of the spine (Figure 7). This suggests the possibility that the spine neck diameter functions as an independent geometrical switch to support physiological states of individual spines, such as growth, stability, or atrophy.

Limitations in spine group segmentation

Despite its overall promise, our algorithm encounters challenges in segmenting spines with complex morphologies and spatial arrangements. Below, we discuss three key failure cases, illustrated in Figure 8. The consistently high precision suggests that, when a spine is detected, it is almost always correct (few false positives). However, the moderate recall for IoU alone indicates that some spines are missed (false negatives), potentially affecting downstream morphological analyses. The substantial performance improvement with union-based IoU suggests that a more inclusive segmentation strategy enhances detection reliability.

Dendritic spines frequently appear in dense clusters, complicating their segmentation. These spine groups are two or more spines whose vertices are directly connected, and, after the shaft is removed, they still group together. Figure 8 shows a region containing tightly packed spines where the algorithm struggles to distinguish individual structures due to overlapping curvature features. This limitation highlights the need for improved clustering-based segmentation techniques or the integration of additional geometric descriptors capable of differentiating individual spines in high-density regions.

Misclassification of dendritic shaft regions as spines

Another failure mode involves the erroneous classification of dendritic shaft regions as spines, as depicted in Figure

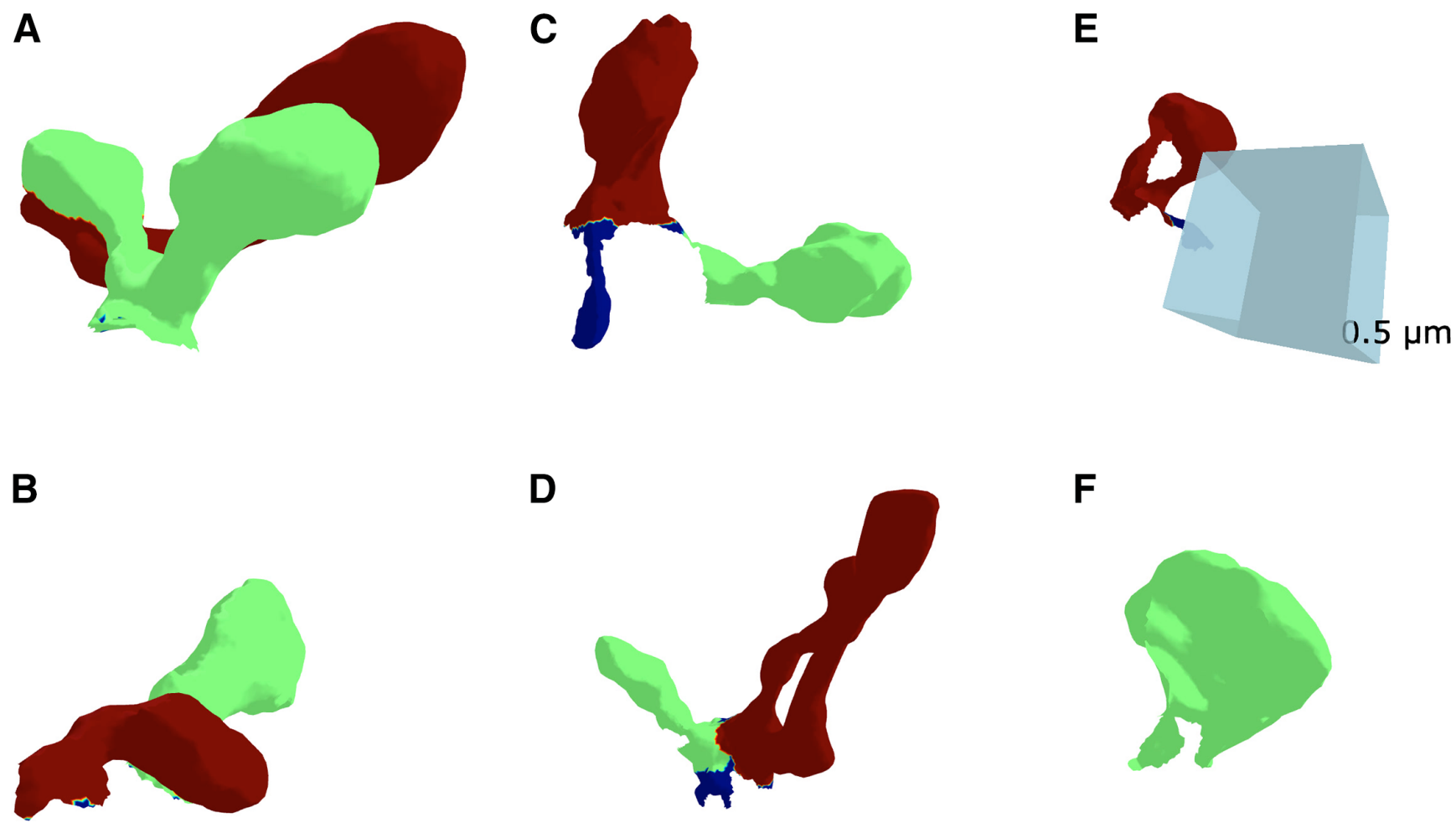

Figure 8. Examples of misclassification encountered during spine segmentation. Blue indicates the shaft mesh, while other colors indicate spine meshes that were misclassified. (A–D) Cases where multiple spines were incorrectly merged and classified as a single spine. (E) A region of the dendritic shaft that was mistakenly labeled as a spine. (F) A fraction of a spine that was only partially labeled. These examples highlight the challenges of accurately separating spines in dense clusters. The triangular mesh is obtained from CA1 dendritic reconstructions.[28–30]

8F. This misclassification likely stems from local curvature variations that resemble spine-like features. To mitigate this issue, we propose the incorporation of spatial continuity constraints, contextual neighborhood information, or multi-scale curvature analysis to better distinguish dendritic shafts from true spine structures.

Scalability and large-dataset limitations

A further limitation arises from the scale of the datasets used in training and evaluation. High-resolution 3D EM reconstructions of dendritic segments generate extremely large meshes, often containing millions of vertices. Processing such datasets requires substantial computational resources, both in terms of memory and runtime. This constraint limits the feasibility of applying our method to very large-scale reconstructions or to entire brain regions without significant preprocessing or downsampling. Moreover, the need to balance mesh resolution with computational efficiency may lead to the loss of fine structural details, particularly in thin spine necks or small protrusions. Addressing this limitation will require the development of more efficient algorithms, parallelized implementations, or hierarchical multi-resolution approaches that can scale to increasingly large datasets while preserving biologically relevant detail.

Outlook

Overall, our findings highlight the potential of feature-enriched geometric learning for robust dendritic spine detection. By improving segmentation accuracy, computational efficiency, and scalability, these models provide a strong foundation for downstream morphological analyses and quantitative studies of synaptic connectivity. Continued refinement of geometric descriptors, multi-resolution strategies, and post-processing methods will further enhance the reliability and applicability of this framework to increasingly large and complex neural reconstructions.

By automating the segmentation of dendritic spines using a deep-learning approach specialized to the distinctive geometries of dendritic spines, this work makes it possible to analyze data at greater scale, setting the stage for future research on the relationship between dendritic spine form and function. In future work, we plan to look at spatial correlations between dendritic spine position along the dendritic shaft and to explore changes in spine shape that occur throughout development, including during long-term potentiation[50] and in Parkinson's disease[51] and other disorders.

DATA AVAILABILITY

To make these methods widely accessible, we have posted an open-source code repository on GitHub (GitHub:curvature-based-dendrite-segmentation), with the trained model, training data, and validation data made available in a linked repository (Zenodo: https://doi.org/10.5281/zenodo.19593999).

ACKNOWLEDGMENTS

We acknowledge Myles Joyce for help with spine segmentation. This work was supported by NSF Technology Hub #1707356, NSF NeuroNex2 #2014862, NSF award 2219894, and NIH grants R01MH095980 and R56MH139176 to K.M.H. and NIH grant T32 NS007292 to A.K.A.G. We acknowledge use of the Brandeis High Performance Computing Cluster (HPCC), which is partially supported by the NSF through DMR-MRSEC 2011846 and OAC-1920147.

AUTHOR CONTRIBUTIONS

A.K.A.G. designed research, developed code, performed analysis, made figures, and wrote the manuscript. M.A.C. designed research and wrote the manuscript. K.M.H. designed research and wrote the manuscript. T.G. F. designed research, performed analysis, and wrote the manuscript.

DECLARATION OF INTERESTS

The authors declare no competing interests.

SUPPLEMENTAL CITATION

References 52–66 appear in the supplemental information.

SUPPLEMENTAL INFORMATION

Supplemental information can be found online at https://doi.org/10.1016/j.bpj.2026.06.005.

REFERENCES

1. Kasthuri, N., K. J. Hayworth, …, J. W. Lichtman. 2015. Saturated reconstruction of a volume of neocortex. *Cell.* 162:648–661.
2. Kuwajima, M., J. M. Mendenhall, and K. M. Harris. 2013. Large-volume reconstruction of brain tissue from high-resolution serial section images acquired by SEM-based scanning transmission electron microscopy. *In Nanoimaging*, Methods in Molecular Biology (Clifton, N.J.. A. A. Sousa and M. J. Kruhlak, eds Humana Press, Totowa, NJ, pp. 253–273.
3. Bourne, J., and K. M. Harris. 2007. Do thin spines learn to be mushroom spines that remember? *Curr. Opin. Neurobiol.* 17:381–386.
4. Harris, K. M., and S. B. Kater. 1994. Dendritic spines: cellular specializations imparting both stability and flexibility to synaptic function. *Annu. Rev. Neurosci.* 17:341–371.
5. Kandel, E. R., Y. Dudai, and M. R. Mayford. 2014. The molecular and systems biology of memory. *Cell.* 157:163–186.
6. Nelson, S. 2008. Pyramidal neurons: dendritic structure and synaptic integration. *Nat. Rev. Neurosci.* 9:206–221.
7. Yuste, R. 2011. Dendritic spines and distributed circuits. *Neuron.* 71:772–781.
8. Ofer, N., and O. Shefi. 2016. Axonal geometry as a tool for modulating firing patterns. *Appl. Math. Model.* 40:3175–3184.
9. Ofer, N., D. R. Berger, …, R. Yuste. 2021. Ultrastructural analysis of dendritic spine necks reveals a continuum of spine morphologies. *Dev. Neurobiol.* 81:746–757.
10. Ofer, N., R. Benavides-Piccione, …, R. Yuste. 2022. Structural analysis of human and mouse dendritic spines reveals a morphological continuum and differences across ages and species. *eNeuro.* 9.
11. Fiala, J. C., J. Spacek, and K. M. Harris. 2002. Dendritic spine pathology: cause or consequence of neurological disorders? *Brain Res. Rev.* 39:29–54.
12. Merino-Serrais, P., R. Benavides-Piccione, …, J. DeFelipe. 2013. The influence of phospho-tau on dendritic spines of cortical pyramidal neurons in patients with alzheimer's disease. *Brain.* 136:1913–1928.
13. Yu, W., and B. Lu. 2012. Synapses and dendritic spines as pathogenic targets in alzheimer's disease. *Neural Plast.* 2012:1–8.
14. Basu, S., P. K. Saha, …, J. Wlodarczyk. 2018. Quantitative 3-D morphometric analysis of individual dendritic spines. *Sci. Rep.* 8:3545.
15. Pchitskaya, E., P. Vasiliev, …, I. Bezprozvanny. 2023. SpineTool is an open-source software for analysis of morphology of dendritic spines. *Sci. Rep.* 13:10561.
16. Bernal-Garcia, S., A. P. Schlotter, …, L. A. Hammond. 2025. A deep learning pipeline for accurate and automated restoration, segmentation, and quantification of dendritic spines. *Cell Rep. Methods.* 5:101179.
17. Smirnov, M. S., T. R. Garrett, and R. Yasuda. 2018. An open-source tool for analysis and automatic identification of dendritic spines using machine learning. *PLoS One.* 13:e0199589.
18. Su, R., C. Sun, …, T. D. Pham. 2014. A novel method for dendritic spines detection based on directional morphological filter and shortest path. *Comput. Med. Imaging Graph.* 38:793–802.
19. Ustinova, A., E. Volkova, …, E. Pchitskaya. 2024. Generate and analyze three-dimensional dendritic spine morphology datasets with SpineTool software. *Curr. Protoc.* 4:e70061.
20. Vidaurre-Gallart, I., I. Fernaud-Espinosa, …, M. García-Lorenzo. 2022. A deep learning-based workflow for dendritic spine segmentation. *Front. Neuroanat.* 16:2022.
21. Vogel, F. W., S. Alipek, …, M. Kaschube. 2023. Utilizing 2d-region-based CNNs for automatic dendritic spine detection in 3D live cell imaging. *Sci. Rep.* 13:20497.
22. Xiao, X., M. Djurisic, …, D. L. Rubin. 2018. Automated dendritic spine detection using convolutional neural networks on maximum intensity projected microscopic volumes. *J. Neurosci. Methods.* 309:25–34.
23. Al-Khater, W., and S. Al-Madeed. 2024. Using 3d-vgg-16 and 3d-resnet-18 deep learning models and fabemd techniques in the detection of malware. *Alex. Eng. J.* 89:39–52.
24. Çiçek, Ö., A. Ahmed, …, O. Ronneberger. 2016. 3d u-net: Learning dense volumetric segmentation from sparse annotation. *In* Medical Image Computing and Computer-Assisted Intervention – MICCAI 2016. S. Ourselin, L. Joskowicz, and …, W. Wellseds Springer International Publishing, Cham, pp. 424–432.
25. Maturana, D., and S. Scherer. 2015. Voxnet: A 3d convolutional neural network for real-time object recognition. *In* 2015 IEEE/RSJ International Conference on Intelligent Robots and Systems (IROS) IEEE, pp. 922–928.
26. Bourne, J. N., and K. M. Harris. 2008. Balancing structure and function at hippocampal dendritic spines. *Annu. Rev. Neurosci.* 31:47–67.
27. Wu, C.-H., T. G. Fai, …, C. S. Peskin. 2015. Simulation of osmotic swelling by the stochastic immersed boundary method. *SIAM J. Sci. Comput.* 37:B660–B688.
28. Bourne, J. N., and K. M. Harris. 2011. Coordination of size and number of excitatory and inhibitory synapses results in a balanced structural plasticity along mature hippocampal ca1 dendrites during ltp. *Hippocampus.* 21:354–373.
29. Bromer, C., T. M. Bartol, …, K. M. Harris. 2018. Long-term potentiation expands information content of hippocampal dentate gyrus synapses. *Proc. Natl. Acad. Sci. USA.* 115:E2410–E2418.
30. Chirillo, M. A., M. S. Waters, …, K. M. Harris. 2019. Local resources of polyribosomes and ser promote synapse enlargement and spine clustering after long-term potentiation in adult rat hippocampus. *Sci. Rep.* 9:3861.
31. Raissi, M., P. Perdikaris, and G. E. Karniadakis. 2019. Physics-informed neural networks: A deep learning framework for solving forward and inverse problems involving nonlinear partial differential equations. *J. Comput. Phys.* 378:686–707.
32. George, K., Y. Kevrekidis, …, L. Yang. 2021. Physics-informed machine learning. *Nat. Rev. Phys.* 3:1–19.
33. Lu, L., X. Meng, …, G. E. Karniadakis. 2021. Deepxde: A deep learning library for solving differential equations. *SIAM Rev.* 63:208–228.
34. Samaniego, E., C. Anitescu, …, T. Rabczuk. 2020. An energy approach to the solution of partial differential equations in computational mechanics via machine learning: Concepts, implementation and applications. *Comput. Methods Appl. Mech. Eng.* 362:112790.
35. Rao, C., H. Sun, and Y. Liu. 2020. Physics-informed deep learning for incompressible laminar flows. *Theor. Appl. Mech. Lett.* 10:207–212.
36. Li, H., Z. Zhang, …, X. Si. 2024. A review on physics-informed data-driven remaining useful life prediction: Challenges and opportunities. *Mech. Syst. Signal Process.* 209:111120.
37. Hornik, K., M. Stinchcombe, and H. White. 1989. Multilayer feedforward networks are universal approximators. *Neural Netw.* 2:359–366.
38. LeCun, Y., Y. Bengio, and G. Hinton. 2015. Deep learning. *Nature.* 521:436–444.
39. Lee, T. C., R. L. Kashyap, and C. N. Chu. 1994. Building skeleton models via 3-d medial surface axis thinning algorithms. *CVGIP Graph. Models Image Process.* 56:462–478.
40. Zhang, T. Y., and C. Y. Suen. 1984. A fast parallel algorithm for thinning digital patterns. *Commun. ACM.* 27:236–239.

41. Cignoni, P., M. Callieri, …, G. Ranzuglia. 2008. Meshlab: an open-source mesh processing tool. *In* Eurographics Italian chapter conference. V. Scarano, R. De Chiara, and U. Erra, eds, pp. 129–136.
42. Edelsbrunner, H., D. Kirkpatrick, and R. Seidel. 1983. On the shape of a set of points in the plane. *IEEE Trans. Inf. Theory.* 29:551–559.
43. Harris, K. M., J. Spacek, …, R. Burns. 2015. A resource from 3d electron microscopy of hippocampal neuropil for user training and tool development. *Sci. Data.* 2:150046.
44. Bartol, T. M., C. Bromer, …, T. J. Sejnowski. 2015. Nanoconnectomic upper bound on the variability of synaptic plasticity. *eLife.* 4:e10778.
45. Virtanen, P., R. Gommers, …, Y. Vázquez-Baeza. 2020. SciPy 1.0: fundamental algorithms for scientific computing in Python. *Nat. Methods.* 17:261–272.
46. Dierckx, P. 1982. Algorithms for smoothing data with periodic and parametric splines. *Comput. Graph. Image Process.* 20:171–184.
47. Dierckx, P. 1995. Curve and Surface Fitting with Splines. Oxford University Press.
48. Fai, T. G., R. Kusters, …, L. Mahadevan. 2017. Active elastohydrodynamics of vesicles in narrow blind constrictions. *Phys. Rev. Fluids.* 2:113601.
49. Park, Y., and T. G. Fai. 2020. Dynamics of vesicles driven into closed constrictions by molecular motors. *Bull. Math. Biol.* 82:141.
50. Yasumatsu, N., M. Matsuzaki, …, H. Kasai. 2008. Principles of long-term dynamics of dendritic spines. *J. Neurosci.* 28:13592–13608.
51. Villalba, R. M., and Y. Smith. 2018. Loss and remodeling of striatal dendritic spines in parkinson's disease: from homeostasis to maladaptive plasticity? *J. Neural Transm.* 125:431–447.
52. Abadi, M., P. Barham, …, X. Zheng. 2016. Tensorflow: a system for large-scale machine learning. *In Proceedings of the 12th USENIX Conference on Operating Systems Design and Implementation*, OSDI'16. USENIX Association, USA, pp. 265–283.
53. Dawson-Haggerty, M. 2019. Trimesh: A python library for loading and using triangular meshes. https://trimesh.org.
54. Garland, M., and P. S. Heckbert. 2023. Surface Simplification Using Quadric Error Metrics, 1 edition. Association for Computing Machinery, New York, NY, USA.
55. Harris, C. R., K. J. Millman, …, T. E. Oliphant. 2020. Array programming with NumPy. *Nature.* 585:357–362.
56. Hoerl, A. E., and R. W. Kennard. 1970. Ridge regression: Biased estimation for nonorthogonal problems. *Technometrics.* 12:55–67.
57. Kazhdan, M., M. Bolitho, and H. Hoppe. 2006. Poisson surface reconstruction. *In Proceedings of the Fourth Eurographics Symposium on Geometry Processing*, SGP '06. K. Polthier and A. Sheffer, eds Eurographics Association, Goslar, DEU, pp. 61–70.
58. Kazhdan, M., and H. Hoppe. 2013. Screened poisson surface reconstruction. *ACM Trans. Graph.* 32:1–13.
59. Kingma, D. K., and J. Ba. 2015. Adam: a method for stochastic optimization. *In* ICLR. Y. Bengio and U. LeCun, eds https://doi.org/10.48550/arXiv.1412.6980.
60. Meyer, M., M. Desbrun, …, H. Alan. 2003. Barr. Discrete differential-geometry operators for triangulated 2-manifolds. *In* Visualization and Mathematics III. H.-C. Hege and K. Polthier, eds Springer Berlin Heidelberg, Berlin, Heidelberg, pp. 35–57.
61. O'Neill, B. 1997. Elementary Differential Geometry. Academic Press.
62. Sullivan, J. M. 2006. Curvature measures for discrete surfaces. *In* ACM SIGGRAPH 2006 Courses ACM, pp. 10–13.
63. Tibshirani, R. 1996. Regression shrinkage and selection via the lasso. *Journal of the royal statistical society series b-methodological.* 58:267–288.
64. van der Walt, S., J. L. Schönberger, …, T. Yu. 2014. scikit-image contributors. scikit-image: image processing in python. *PeerJ.* 2:e453.
65. Yuksel, C. 2015. Sample elimination for generating poisson disk sample sets. *Comput. Graph. Forum.* 34:25–32.
66. Zhou, Q.-Y., J. Park, and V. Koltun. 2018. Open3D: A modern library for 3D data processing. *arXiv.* https://doi.org/10.48550/arXiv.1801.09847.

Biophysical Journal, Volume *125*

Supplemental information

Curvature-based machine-learning method for automated segmentation of dendritic spines

Abdel Kader A. Geraldo, Michael A. Chirillo, Kristen M. Harris, and Thomas G. Fai

Supplemental Information

Curvature-based Machine Learning Method for Automated Segmentation of Dendritic Spines. Abdel Kader A. Geraldo[†], Michael A. Chirillo[§], Kristen M. Harris[‡], Thomas G. Fai*

[†]Department of Mathematics, Brandeis University, Waltham, MA, USA
[§]Department of Biological Sciences, University of Rhode Island, Kingston, RI, USA
[‡]Department of Neuroscience, University of Texas at Austin, Austin, TX, USA
*Department of Mathematics and Volen Center for Complex Systems, Brandeis University, Waltham, MA, USA tfai@brandeis.edu

Supplemental Materials and Methods

S1. Enhancement of Curvature through Image Processing

For the first panel of Figure 3 of the Main Text, we choose parameters that emphasize the neck region of the triangular dendritic mesh. In particular, we visualize the function

$$F = \widetilde{\mathbf{H}} + \widetilde{\mathbf{K}} \qquad \begin{cases} a_{\mathbf{H}} & = 5 \\ b_{\mathbf{H}} & = 10 \\ a_{\mathbf{K}} & = -1 \\ b_{\mathbf{K}} & = -10 \end{cases} .$$

This combination enhances the contrast of the neck surface and provides an interpretable geometric baseline for comparison with the learned probabilities.

S2. Training Details

The DNN architecture was built using `TensorFlow` (v2.16.2) [1], and training was performed for more than 1500 epochs for each model, after which the loss had converged. We trained the network using the Adam optimizer [2] with a piecewise constant learning rate schedule. The learning rate was set to 10^{-2} for the first 1000 training steps, reduced to 10^{-3} for steps 1000–3000, and further reduced to 5×10^{-4} for the remainder of training. Only a single batch was used per training step, corresponding to a batch size of 1, because each dendritic mesh is processed independently and cannot be meaningfully combined with others within the same batch.

L1 and L2 regularization were applied to the hidden layers to improve generalization and reduce overfitting. This dual regularization strategy encourages sparsity in the learned weights while penalizing large parameter values [3, 4], thereby improving robustness across diverse inputs.

The class imbalance between shaft and spine vertices was handled using class weights derived from the inverse log-frequency of each class. Let c_k denote the number of vertices belonging to class k and $T = \sum_k c_k$ the total number of vertices. The unnormalized class weight is

$$w'_k = \max\left(\log\left(\frac{2T}{c_k}\right), 1\right),$$

and the final normalized weights were obtained by $w_k = w'_k / \sum_j w'_j$. These weights were applied to the cross-entropy loss during training to prevent the model from being biased toward the majority class.

S3. Computer Specifics

All geometric models (DNN_1, DNN_2, DNN_3) were trained on an HPCC CPU-only node equipped with 2×Intel Cascade Lake CPUs (40 total cores) and 754 GB of RAM. The CNN-based models (U-Net, VoxNet, VGG16–FCN) could not be trained on a local machine due to their substantially higher computational and memory requirements. Even after downsampling the meshes to 5,000 vertices, the CNN architectures exceeded the memory capacity of the local system and were therefore trained exclusively on the HPCC node.

All testing and inference experiments were performed on a MacBook Pro equipped with an Apple M4 Max chip and 64 GB of unified memory.

The geometric models run an order of magnitude faster than the CNN baselines, with DNN_3 achieving the lowest runtime (43–45 s/iteration), while the CNN models require 5–8 times longer per iteration despite comparable memory usage. Table S2 summarizes the runtime and memory characteristics of all evaluated models.

S4. Performance Metrics

Various performance metrics were selected to evaluate the quality of the predicted segmentations. The DICE coefficient, Jaccard index (IoU), and AUC provide complementary perspectives on segmentation performance. The DICE coefficient quantifies the overlap between the predicted region P and the ground-truth region G, and is defined as

$$\text{DICE} = \frac{2|P \cap G|}{|P| + |G|}.$$

Higher values indicate stronger agreement between prediction and ground truth. The Jaccard index (IoU) offers a stricter measure of overlap, computed as

$$\text{IoU} = \frac{|P \cap G|}{|P \cup G|},$$

and penalizes both false positives and false negatives. Finally, the AUC (Area Under the ROC Curve) evaluates the model's ability to discriminate between classes across all possible thresholds; the ROC (Receiver Operating Characteristic) curve plots the true-positive rate against the false-positive rate. Together, these metrics capture spatial accuracy, overlap quality, and classification discriminability, providing a comprehensive assessment of segmentation performance.

S5. Discrete Gaussian and Mean Curvature

In this section, we briefly review the discrete differential geometry formulation presented in [5–8] to derive the Gaussian and the mean curvature of triangular meshes. Assume the mesh has $\mathcal{V}$ vertices and $\mathcal{T}$ triangular faces. Each triangular face $\mathcal{T}_l \in \mathcal{T}$ has vertices denoted by k_i^l, k_{i+1}^l, and k_{i+2}^l. These vertices are indexed in a counterclockwise order around the face, defined as follows:

Let $k_{\text{next}}(\cdot)$ denote the next vertex in the counterclockwise direction from $\cdot$. Then:

- k_i^l is the first vertex.
- $k_{i+1}^l = k_{\text{next}}(k_i^l)$ is the next vertex in the counterclockwise direction.
- $k_{i+2}^l = k_{\text{next}}(k_{i+1}^l)$ is the vertex following k_{i+1}^l in the counterclockwise direction. Then $k_{i+2}^l = k_{\text{next}}(k_{\text{next}}(k_i^l))$.
- $k_i^l = k_{\text{next}}(k_{i+2}^l) = k_{\text{next}}(k_{\text{next}}(k_{\text{next}}(k_i^l)))$ completes the cycle.

This leads to the vertex indices cycling according to:

$$k_i^l = k_{(i-1 \mod 3)+1}^l,$$

where $(i \mod 3) + 1$ cycles through the indices $\{1, 2, 3\}$. Moreover, we will denote the set of indices of the 1-ring neighbors of a vertex with index k by $\mathcal{N}_k$. This set includes the indices of the vertices whose edges are connected to the vertex $\mathbf{X}_k$:

$$\mathcal{N}_k = \{j \mid \text{there exists an edge between } \mathbf{X}_k \text{ and } \mathbf{X}_j\}.$$

S5.1. **Discrete Gaussian Curvature.** Let us consider an infinitesimal area $\mathcal{A}$ and denote its diameter by diam($\mathcal{A}$). Additionally, let $\mathcal{A}^{\mathcal{G}}$ denote the area of the image of the Gauss map associated with $\mathcal{A}$. We can express the discrete Gaussian curvature, $\widehat{\kappa}_G$ at a vertex $\mathbf{X}_i$ as:

$$\widehat{\kappa}_G = \frac{1}{\mathcal{A}} \int\int_{\mathcal{A}} \kappa_G dA = \frac{1}{\mathcal{A}} \sum_{i \in \mathcal{A}} \mathbf{K}_i, \text{ with } \mathbf{K}_i = 2\pi - \sum_{j \in \mathcal{N}_i} \theta_j \tag{1}$$

where θ_j are the interior angles at $\mathbf{X}_i$ of the triangles meeting there. The term $\mathbf{K}_i$ is known as the defect angle at vertex $\mathbf{X}_i$. Considering vertices $\mathbf{X}_j$ in the 1-ring neighborhood of $\mathbf{X}_i$, see Fig S1, the angle θ_j can be computed as:

$$\cos\theta_j = \frac{(\mathbf{X}_{j-1} - \mathbf{X}_i) \cdot (\mathbf{X}_j - \mathbf{X}_i)}{\|(\mathbf{X}_{j-1} - \mathbf{X}_i)\| \cdot \|(\mathbf{X}_j - \mathbf{X}_i)\|} = \frac{\mathbf{E}_{i,j-1} \cdot \mathbf{E}_{ij}}{\|\mathbf{E}_{i,j-1}\| \cdot \|\mathbf{E}_{ij}\|},$$

where $\mathbf{E}_{i,j} = \mathbf{X}_j - \mathbf{X}_i$ is the edge of the vertices $\mathbf{X}_i, \mathbf{X}_j$.

S5.2. **Mean curvature.** The discrete mean curvature, $\widehat{\kappa}_H$, at a vertex i is defined as:

$$\widehat{\kappa}_H = \frac{1}{\mathcal{A}} \int\int_{\mathcal{A}} \kappa_H dA = \frac{1}{\mathcal{A}} \sum_{i \in \mathcal{A}} \mathbf{H}_i, \qquad 2\mathbf{H}_i = \int\int_{\mathcal{A}} \kappa_H dA. \tag{2}$$

To compute $\mathbf{H}_i$, we consider two equivalent methods [5–8]. First, assuming vertices adjacent to i in cyclic order are $\mathbf{X}_j, \mathbf{X}_{j+1}, \ldots \mathbf{X}_{j+n}$. we have:

$$\begin{aligned} 2\mathbf{H}_i &= \sum_{j \in \mathcal{N}_i} \mathbf{E}_{i,j} \times \mathbf{n}_{j+1} - \mathbf{E}_{i,j} \times \mathbf{n}_j \\ &= - \sum_{j \in \mathcal{N}_i} \mathbf{E}_{j,j-1} \times \mathbf{n}_j, \end{aligned}$$

where $\mathbf{n}_j$ is the normal vector to the triangle with vertices $\mathbf{X}_i, \mathbf{X}_{j-1}, \mathbf{X}_j$:

$$\mathbf{n}_j = \frac{(\mathbf{X}_{j-1} - \mathbf{X}_i) \times (\mathbf{X}_j - \mathbf{X}_i)}{\|(\mathbf{X}_{j-1} - \mathbf{X}_i) \times (\mathbf{X}_j - \mathbf{X}_i)\|} = \frac{\mathbf{E}_{i,j-1} \times \mathbf{E}_{i,j}}{\|\mathbf{E}_{i,j-1} \times \mathbf{E}_{i,j}\|}.$$

Alternatively, the second method involves the integral of the Laplace-Beltrami operator [6]:

$$2\mathbf{H}_i = \sum_{j \in \mathcal{N}_i} (\cot\alpha_{i,j} + \cot\beta_{i,j})(\mathbf{X}_i - \mathbf{X}_j),$$

where $\alpha_{i,j}$ and $\beta_{i,j}$ are the angles opposite edge $\mathbf{E}_{i,j}$ in the two incident triangles, as shown in Fig S1.

S5.3. **Vector Area.** In this section, we compute the area $\mathcal{A}$ as in (1), (2), the sum of all the regions that contain vertex $\mathbf{X}_i$,

$$\mathcal{A} = \sum_{i \in \mathcal{A}} A_i.$$

To do this, A_i is computed using either the conic area or the barycentric formula that we will describe below. Both methods are equivalent in any case [6].

S5.3.1. *Conic area.* The formula used to compute the vector area is:

$$\mathbf{A} = \frac{1}{2}\int\int_{\mathcal{A}} \mathbf{n} dA,$$

and specifically in the triangle with edge $\mathbf{E}_i, \mathbf{E}_j$ case, the area is given by:

$$A_{ij} = \frac{1}{2}\|\mathbf{E}_i \times \mathbf{E}_j\|$$

As the conic area is equal to the third of the area in the 1-ring neighborhood of vertex $\mathbf{X}_i$, we have:

$$A_i = \frac{1}{3}A = \frac{1}{6}\sum_{j\in\mathcal{N}_i}\|\mathbf{E}_{i,j-1} \times \mathbf{E}_{ij}\|$$

S5.3.2. *Barycenter Area.* To compute the barycentric area (represented in blue in Figure S1), we first find the centroid $\mathbf{C}_{ij}$ of each triangle in the 1-ring neighborhood:

$$\mathbf{C}_{ij} = \frac{1}{3}(\mathbf{X}_i + \mathbf{X}_{j-1} + \mathbf{X}_j).$$

Next, we compute the middle point $\mathbf{X}_{ij}$ of each edge $\mathbf{X}_i, \mathbf{X}_j$:

$$\mathbf{M}_{ij} = \frac{1}{2}(\mathbf{X}_i + \mathbf{X}_j).$$

Then the barycentric area corresponding to the triangle with the vertices $\mathbf{X}_i, \mathbf{X}_{j-1}, \mathbf{X}_j$ is

$$A_{ij} = \frac{1}{2}\|(\mathbf{M}_{i,j-1} - \mathbf{C}_{ij}) \times (\mathbf{X}_i - \mathbf{C}_{ij})\| + \frac{1}{2}\|(\mathbf{M}_{ij} - \mathbf{C}_{ij}) \times (\mathbf{X}_i - \mathbf{C}_{ij})\|.$$

Thus, the barycentric area of the 1-ring neighborhood A_i is:

$$A_i = \sum_{j\in\mathcal{N}_i} A_{ij}.$$

S6. Skeletonization

In this section, we describe the skeletonization algorithm applied to a 3D mesh using Python packages such as `trimesh`, `open3d`, and `scikit-image`. All experiments were performed in a Python 3.9.6 environment with the following package versions: `numpy` (v2.2.6) [9], `open3d` (v0.18.0) [10], `trimesh` (v4.6.8) [11], and `scikit-image` (v0.24.0) [12]. The goal of this algorithm is to extract a simplified, one-voxel-wide medial axis from a complex dendritic mesh, which can then be used for further geometric analysis and as input to the DNN.
To perform skeletonization, the first step is to ensure that the mesh is watertight. A watertight mesh is a closed surface with no gaps, holes, or disconnected edges, which guarantees a well-defined interior and exterior. If the input mesh is not watertight, we apply a wrapping procedure based on Poisson surface reconstruction to generate a closed representation. Once the watertight mesh is obtained, the pipeline proceeds through mesh simplification, voxelization, skeleton extraction, and vertex-to-skeleton mapping.

S6.1. **Mesh Wrapping.** To ensure the mesh is watertight and suitable for skeletonization, we apply a wrapping procedure based on Poisson surface reconstruction [10, 13, 14]. This step converts the input mesh into a uniformly sampled point cloud, estimates and orients normals, and then reconstructs a closed surface. The implementation is shown below:

```
mesh = trimesh.Trimesh(vertices=vertices, faces=faces)
o3d_mesh = o3d.geometry.TriangleMesh()
o3d_mesh.vertices = o3d.utility.Vector3dVector(mesh.vertices)
o3d_mesh.triangles = o3d.utility.Vector3iVector(mesh.faces)

# Sample points uniformly from the surface
pcd = o3d_mesh.sample_points_poisson_disk(
    number_of_points=number_of_points
)

# Estimate and orient normals
pcd.estimate_normals(
    search_param=o3d.geometry.KDTreeSearchParamHybrid(
        radius=radius, max_nn=max_nn
    )
)
pcd.orient_normals_consistent_tangent_plane(k=10)

# Reconstruct a watertight mesh using Poisson surface reconstruction
mesh_poisson, _ = o3d.geometry.TriangleMesh.create_from_point_cloud_poisson(
    pcd, depth=8
)

vertices = np.asarray(mesh_poisson.vertices)
faces = np.asarray(mesh_poisson.triangles)
```

Here, we first convert the input vertices and faces into a `trimesh.Trimesh` object and then into an Open3D `TriangleMesh`. From this mesh, we generate a uniformly sampled point cloud using Poisson disk sampling [10, 15] Normals are estimated and consistently oriented to ensure correct surface reconstruction. Finally, Poisson surface reconstruction produces a watertight mesh, which is returned as arrays of vertices and faces for subsequent processing. To provide an alternative geometry-preserving option, we also include an implementation of the `alpha-wrap` method, which is based on the classical α-shape formulation [16] and implemented in `PyMeshLab` [17]. This approach generates a watertight surface by constructing an α-shape envelope around the input mesh. The corresponding code is shown below:

```
ms = pymeshlab.MeshSet()
ms.add_mesh(
    pymeshlab.Mesh(
        vertex_matrix=vertices,
        face_matrix=faces
    )
)
```

```
# Apply alpha-wrap reconstruction
ms.apply_filter(
    'generate_alpha_wrap',
    alpha=pymeshlab.PercentageValue(alpha_fraction),
    offset=pymeshlab.PercentageValue(offset_fraction)
)

mesh_wrap = ms.current_mesh()
vertices_wrap = mesh_wrap.vertex_matrix()
faces_wrap = mesh_wrap.face_matrix()
```

Here, the input vertices and faces are loaded into a `MeshSet`, and the `generate_alpha_wrap` filter constructs a watertight surface based on the specified `alpha` and `offset` parameters. The resulting mesh is then extracted as arrays of wrapped vertices and faces for downstream processing.

S6.2. **Mesh Simplification.** We apply a mesh simplification algorithm to reduce the size of the mesh in cases where the number of vertices is very large. This step is crucial for lowering computational complexity and removing unnecessary geometric detail that may interfere with accurate skeletonization. The simplification is performed using quadric decimation [10, 18] in Open3D, as shown in the following code:

```
mesh = trimesh.Trimesh(vertices=vertices, faces=faces)
o3d_mesh = o3d.geometry.TriangleMesh()
o3d_mesh.vertices = o3d.utility.Vector3dVector(mesh.vertices)
o3d_mesh.triangles = o3d.utility.Vector3iVector(mesh.faces)

# Simplify the mesh using quadric decimation
o3d_mesh = o3d_mesh.simplify_quadric_decimation(
    target_number_of_triangles=target_number_of_triangles
)

# Convert back to a trimesh object for compatibility
mesh = trimesh.Trimesh(
    vertices=np.asarray(o3d_mesh.vertices),
    faces=np.asarray(o3d_mesh.triangles)
)
```

Here, we first create a `trimesh.Trimesh` object from the input vertices and faces. This mesh is then converted into an Open3D `TriangleMesh`, which supports advanced mesh processing operations. The `simplify_quadric_decimation` method reduces the number of triangles while preserving the overall geometry and removing fine details. Finally, the simplified mesh is converted back into a `trimesh` object for compatibility with subsequent steps in the pipeline.

S6.3. **Voxelization.** Next, we voxelize the simplified mesh to convert it into a discrete 3D grid representation. This step enables morphological operations such as thinning and skeletonization. The voxelization process is controlled by a resolution parameter, which determines the granularity of the voxel grid:

```
pitch = np.median(mesh.edges_unique_length) * 0.25
```

```
voxelized = mesh.voxelized(pitch=pitch)
filled = voxelized.fill(method="orthogonal")
voxels = filled.matrix.astype(bool)
```

The parameter `pitch` in `mesh.voxelized` is determined from the median edge length of the mesh, scaled by a factor of 0.25. This adaptive choice ties the voxel size to the geometric detail of the mesh, ensuring that the discretization captures fine structures without producing an excessively large grid. The `fill()` method is then applied to close internal cavities, yielding a watertight solid volume. Finally, the voxel grid is converted into a boolean array, where each entry indicates whether a voxel is occupied, providing a suitable representation for subsequent skeletonization.

S7. Spline-Based Interpolation

Given the set of spine vertices, we apply the `splprep` function from the `scipy.interpolate` module in `scipy`(v0.24.0) [19], which computes a B-spline representation of an N-dimensional parametric curve. This enables smoothing and interpolation along the dendritic spine skeleton [20, 21]. The spline is then evaluated using `splev` to obtain a dense set of interpolated points along the curve:

```
points = spine_vertices.T
tck, u = splprep([points[0], points[1], points[2]],
                  s=spline_smooth,
                  k=max(1, min(3, len(points[0]) - 1)))
x_fine, y_fine, z_fine = splev(np.linspace(0, 1, line_num_points), tck)
interpolated_vertices = np.column_stack([x_fine, y_fine, z_fine])
```

Here, `line_num_points` controls the density of interpolation; in our experiments we set it to 150, and the smoothing factor `spline_smooth` was chosen as 0.03. The resulting interpolated vertices are ordered sequentially along the spline, which is ensured by the spline parameterization itself.

S8. Confusion Matrix Analysis

We assess the performance of our model by computing standard classification metrics, including accuracy, precision, recall, and the F1-score. This section details the computation process.
To begin, we illustrate the confusion matrix, which provides a detailed comparison between the predicted classifications and the ground truth annotations. This allows for an in-depth evaluation of classification performance. In our framework, a group of vertices is predicted as spine if its IoU with the annotated spine vertices it overlaps exceeds 0.7.
The classification outcomes are defined as follows:

- **True Positives (TP)**: Spines correctly identified by the algorithm.
- **False Negatives (FN)**: Spines present in the ground truth but missed by the algorithm.
- **False Positives (FP)**: Shaft regions incorrectly classified as spines.
- **True Negatives (TN)**: Shaft regions correctly classified.

Once the confusion matrix is computed, we derive the following performance metrics:

Precision. Precision evaluates the reliability of spine predictions:

$$\text{Precision} = \frac{\text{TP}}{\text{TP} + \text{FP}}.$$

Recall. Recall measures the model's ability to detect actual spines:

$$\text{Recall} = \frac{\text{TP}}{\text{TP} + \text{FN}}.$$

F1-score. The F1-score provides a balanced measure of precision and recall:

$$\text{F1-score} = 2 \times \frac{\text{Precision} \times \text{Recall}}{\text{Precision} + \text{Recall}}.$$

S9. Dendritic Spine Analysis Using 3D CNNs

In this section, we evaluate dendritic spine segmentation using several established 3D convolutional neural network (CNN) architectures, including U-Net [22], VGG16 [23], and the simple VoxNet [24] algorithm, in order to benchmark our proposed method. Because these architectures operate on volumetric data, the original triangular meshes must be converted into 3D voxel grids prior to training. This section describes the preprocessing pipeline used to transform the electron microscopy (EM) triangular meshes into volumetric representations suitable for 3D CNNs.

S9.1. **Preprocessing Triangular EM Meshes into Volumes.** To apply 3D CNN architectures for segmentation, the triangular meshes must first be transformed into structured 3D voxel grids. This requires two main steps: resizing the mesh vertices and voxelizing the mesh into a multi-class 3D grid.

First, we resize each triangular mesh so that the resulting voxel grids remain computationally manageable. Larger meshes lead to significantly higher memory usage and computational cost; therefore, resizing the meshes according to hardware constraints is essential. In our experiments, each mesh is resized to around 5000 vertices. We also explored augmenting the dataset to increase variability, but the associated computational overhead made training prohibitively slow, and we therefore discontinued this approach.

Next, we convert the mesh into a volumetric representation using the `trimesh` voxelization pipeline. The voxelization step discretizes the mesh into a 3D grid, after which we fill the interior of the mesh using the `fill(method="orthogonal")` function. Voxels inside the dendritic branch are assigned label 1, while voxels outside remain 0.

After generating the dendritic branch volume, we construct the ground-truth shaft labels. To do this, we first create an expanded triangular mesh that tightly encloses the true shaft surface, by wrapping procedure based on Poisson surface procedure described in S6.1. We then voxelize and fill this expanded shaft mesh in the same manner as before. Voxels inside this expanded shaft region are assigned label 2. As the expanded mesh fully encloses the true shaft, it ensures that all shaft voxels are correctly captured, even in regions where the original mesh may be thin or irregular.

Finally, the shaft volume (label 2) is overlaid onto the dendritic branch volume (label 1), replacing any overlapping voxels. The resulting multi-class volume contains:

- label 0: background,
- label 1: dendritic spines,
- label 2: dendritic shaft.

Figure S6 depicts the voxel grid of a training dendrite, where three slices of the volume show the voxel grids in the x, y, and z planes. The background is labeled in white, the spines in blue, and the expanded shaft region in red.
This volumetric representation serves as the input to the 3D CNN architectures during training. The learning objective is to correctly identify the shaft voxels (label 2) within the dendritic branch volume. After training, the predicted shaft voxels can be mapped back onto the original triangular mesh to recover the segmented shaft surface.

S9.2. **Training Procedure.** The training procedure for the 3D CNN follows the same general strategy used for the DNN, with the main differences arising from the 3D convolutional architecture. We follow the same procedure described in Section S2. The strong class imbalance between shaft and spine vertices was handled using class weights computed from the inverse log-frequency of each class, which reduces bias toward the majority class and stabilizes training.
The network produces a volumetric prediction in which each voxel contains a vector of probabilities corresponding to the shaft and spine classes. A softmax activation function is applied to obtain voxel-wise class probabilities.
Because the final segmentation is defined on the mesh rather than the voxel grid, each mesh vertex is mapped to its corresponding voxel index. We then retrieve the probability values at that voxel location and use them to assign a class label to the mesh vertex, ensuring consistency between the volumetric CNN output and the surface-based representation of the dendrite. Since the meshes are downsampled before being fed to the CNN, the predicted probabilities are subsequently remapped back to the original high-resolution mesh. This yields the probability matrix $\overline{\mathbf{Y}} = f_\theta(\mathbf{Z}) \in [0,1]^{n\times 2}$, after which spine and shaft detection is restarted following the procedure described in Section 2.4 of the Main Text.

Supplemental Tables

Symbol	Meaning
$\mathbf{X}_l$	Vertex position on dendritic mesh
$\mathbf{E}_{l,m}$	Edge vector between vertices $\mathbf{X}_l$ and $\mathbf{X}_m$
$\mathbf{n}_j$	Triangle normal vector
A	Triangle area
$\mathbf{H}$, $\mathbf{K}$	Mean and Gaussian curvature (smoothed)
$\widetilde{\mathbf{H}}$, $\widetilde{\mathbf{K}}$	Sigmoid-enhanced curvature values
$a_{\mathbf{H}}, b_{\mathbf{H}}$; $a_{\mathbf{K}}, b_{\mathbf{K}}$	Curvature-enhancement parameters
k_{bend}	Bending coefficient in smoothing flow
$\mathbf{W}_{bend}$, $\mathbf{F}_{bend}$	Bending energy and force
$\zeta(x)$	Sigmoid function
$\mathbf{V}_j$	Shaft-skeleton vertex
$\mathbf{D}_l$, $\mathbf{D}$	Distance to nearest skeleton point; distance vector
$\mathbf{S}^k$	K-means region label for k clusters
DNN_1, DNN_2, DNN_3	Deep neural networks for segmentation
$\mathbf{Z}_{i,j}$	Feature vector for vertex i of mesh j
$\mathbf{Y}_{i,j}$	Ground-truth label (shaft/spine)
f_θ	Neural network mapping features to predictions
$\overline{\mathbf{Y}}$	Predicted probability matrix
$\widetilde{\mathcal{X}}_{\text{spine}}$, $\widetilde{\mathcal{X}}_{\text{shaft}}$	Vertices classified as spine / shaft
a, b	Classification threshold parameters
$\mathcal{N}_i$	1-ring neighborhood of vertex $\mathbf{X}_i$
$\mathcal{G}_i$	Connected vertex group containing $\mathbf{X}_i$
$\widetilde{\mathcal{Y}}^i_{\text{part}}$	Connected component of a part type
$\widetilde{\mathcal{Y}}_{\text{shaft}}$	All connected shaft components
$\mathcal{Y}^0_{\text{shaft}}$	Largest shaft component (entire shaft)
$\mathcal{Y}_{\text{spine}}$	Set of all spine components
$\mathcal{Y}^i_{\text{spine}}$	Individual spine component

Table S1. Summary of symbols and variables used in Section 2 of the Main Text.

**Model**	**Runtime (s/Iterations)**	**Peak Memory (GB)**
DNN_1	47–50	0.114
DNN_2	56–58	0.143
DNN_3	43–45	0.314
U-Net	330–350	0.175
VoxNet	290–295	0.189
VGG16–FCN	370–380	0.211

Table S2. Runtime and memory benchmarks for all models. All training was performed on an HPCC CPU-only node with 2×Intel Cascade Lake CPUs (40 cores) and 754 GB RAM. All testing was performed on a MacBook Pro with an Apple M4 Max chip and 64 GB memory.

Category	Description
**Training dataset**	Six high-resolution 3D EM reconstructions of dendritic segments from CA1 hippocampus of P21 rats. Slices received either control stimulation or *in vitro* theta-burst stimulation to induce LTP [25–27].
**Training annotations**	Spines and shafts provided as separate watertight `.obj` meshes. Wrapped into unified dendritic meshes using the method in S6.1.
**Testing dataset**	Nanoconnectomic 3D EM reconstruction of hippocampal neuropil from the axon–spine coupling study [28].
**Testing annotations**	151 meshes available; 28 spiny dendritic branches selected based on annotation consensus. A mesh was included only if at least two annotators agreed on the presence of a spine. Individual spines retained only if labeled by at least two annotations.
**Visualization dataset**	Dendritic segments from the large-scale nanoconnectomic EM reconstruction of mouse neocortex by Kasthuri et al. [29]. This dataset provides densely reconstructed neuropil at nanometer resolution but does not include manual spine or shaft annotations. Used only for qualitative visualization (Figure 6 of the Main Text).
**Visualization usage**	Used solely to demonstrate generalization of the method and to visualize predicted shafts and spines. Not used for training, validation, or quantitative testing.

Table S3. Consolidated summary of datasets, annotations, and selection criteria used for training, testing, and qualitative visualization.

Model	AUC	Criterion	Variant	Precision	Recall	F1-score
**U-Net** (a = 0.05)	0.792	IoU	Standard	0.778	0.697	0.735
			Union	0.792	0.756	0.773
		DICE	Standard	0.798	0.785	0.791
			Union	0.809	0.844	0.826
DNN_3 (a = 13)	0.717	IoU	Standard	0.804	0.794	0.799
			Union	0.818	0.873	0.845
		DICE	Standard	0.811	0.832	0.821
			Union	0.822	0.895	0.857
**Vox Net** (a =10)	0.682	IoU	Standard	0.817	0.311	0.451
			Union	0.811	0.299	0.437
		DICE	Standard	0.840	0.366	0.510
			Union	0.836	0.355	0.498
DNN_2 (a = 0.5)	0.605	IoU	Standard	0.805	0.667	0.729
			Union	0.824	0.754	0.788
		DICE	Standard	0.813	0.702	0.753
			Union	0.833	0.804	0.818
**VGG16 FCN** (a = 16)	0.466	IoU	Standard	0.579	0.202	0.299
			Union	0.610	0.229	0.333
		DICE	Standard	0.680	0.312	0.428
			Union	0.817	0.246	0.378
DNN_1 (a = 3)	0.408	IoU	Standard	0.782	0.654	0.713
			Union	0.790	0.685	0.734
		DICE	Standard	0.803	0.741	0.770
			Union	0.813	0.790	0.801

TABLE S4. Comparison of segmentation performance across geometric-informed models (DNN_1, DNN_2, DNN_3) and established CNN baselines (U-Net, VoxNet, VGG16-FCN). AUC is reported once per model, while Precision, Recall, and F1-score are evaluated under both IoU and DICE criteria, each with Standard and Union variants. Among the CNN baselines, U-Net achieves the highest AUC (0.792) and strong overall performance, whereas VoxNet and VGG16-FCN show moderate and weak performance, respectively. Across all metrics, the geometric-informed model DNN_3 provides the most consistent and accurate segmentation, achieving the highest Recall and F1-scores—particularly under the Union variants, which better capture continuous spine regions. The weighting parameters used in the geometric-informed models are specified in Eq. (6) of the Main Text, where the values of a for each model are listed and the parameter b is fixed at 0.81.

Supplemental Figures

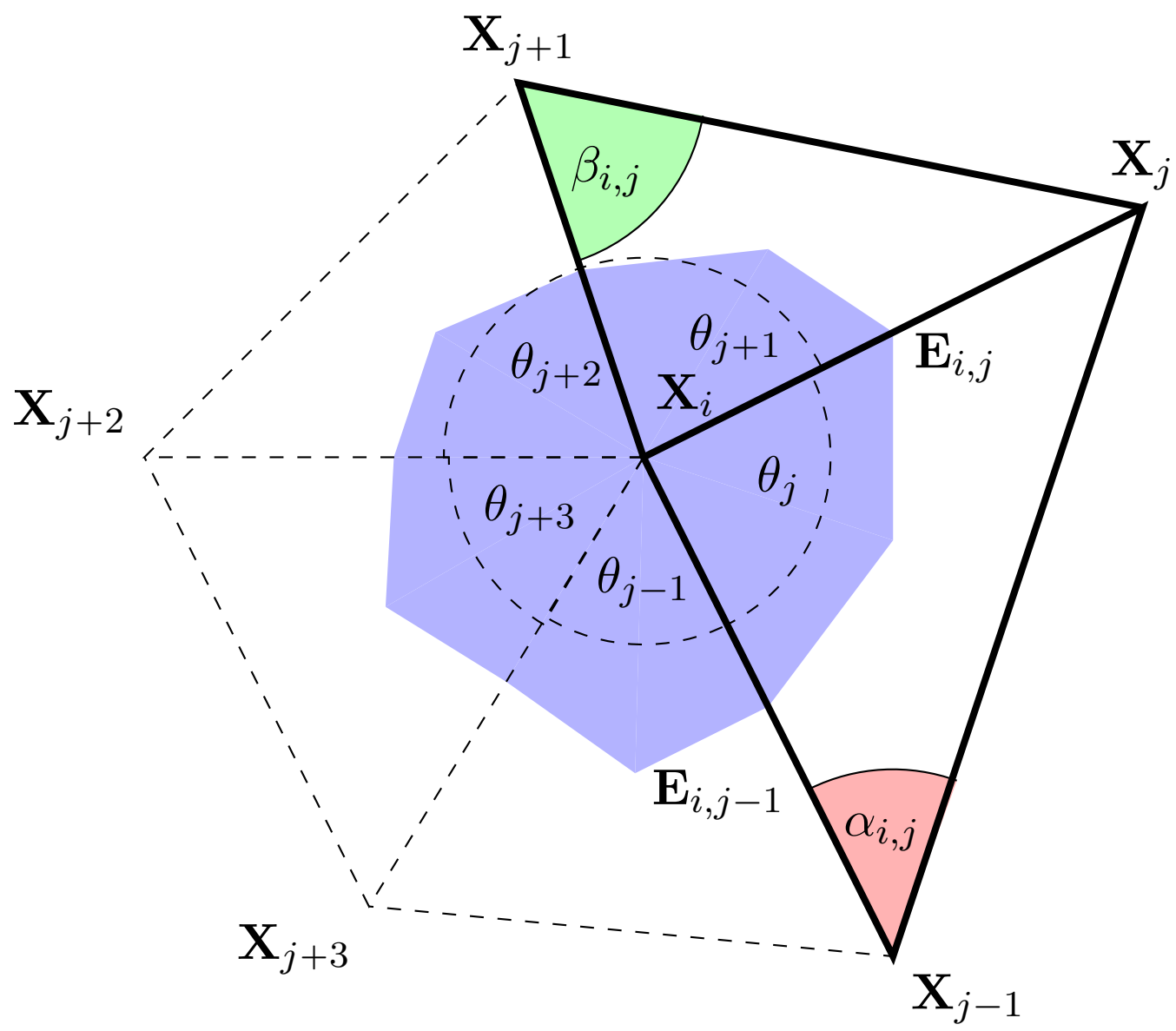

Figure S1. The graph shows the five triangular meshes of 1-ring neighbors to the point $p = \mathbf{X}_i$. It also depicts the incident angles $\alpha_{i,j}$ and $\beta_{i,j}$ opposite to the edge $\mathbf{E}_{i,j} = \mathbf{X}_j - \mathbf{X}_i$. The blue area constitutes the barycentric area A_p.

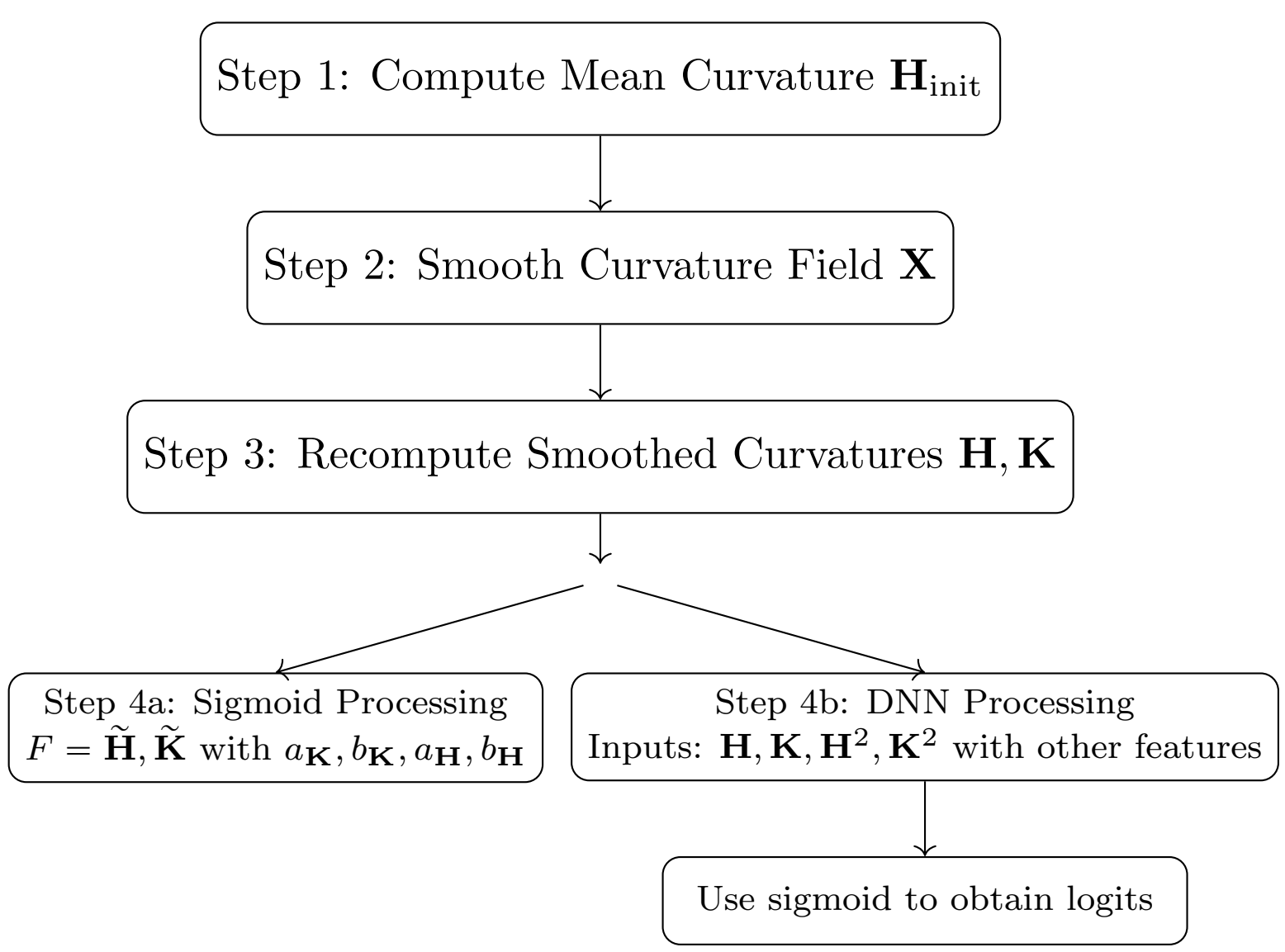

Figure S2. Flowchart of the mesh curvature preprocessing steps.

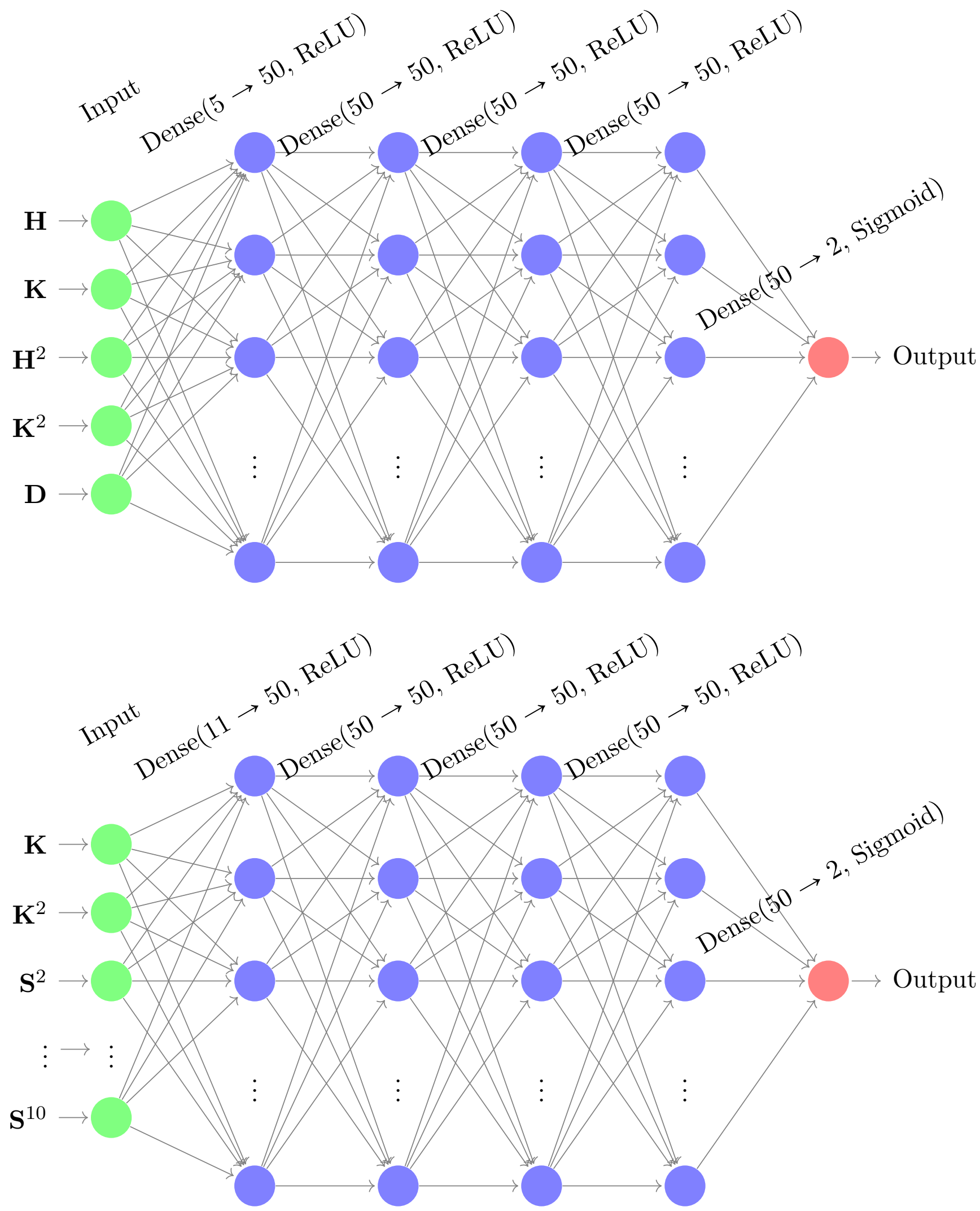

FIGURE S3. The top diagram illustrates the architecture of the deep neural network ($\mathbf{DNN}_2$), which improves upon $\mathbf{DNN}_1$. This model closely resembles the previous network but incorporates an additional input feature: the distance $\mathbf{D}$ between the central curve of the shaft (computed using $\mathbf{DNN}_1$) and the mesh vertices. The output layer employs a sigmoid activation function, consistent with the design of the earlier network. The bottom diagram shows the architecture of the deep neural network ($\mathbf{DNN}_3$) with additional input features. This model extends the previous design by enriching the input layer with multiple geometric and topological descriptors, including the Gaussian curvature and its squared value, as well as segmentation descriptors $\mathbf{S}^k$ obtained from K-means clustering of the shortest distances between mesh vertices and the dendrite skeleton. As in the earlier models, the output layer uses a sigmoid activation function.

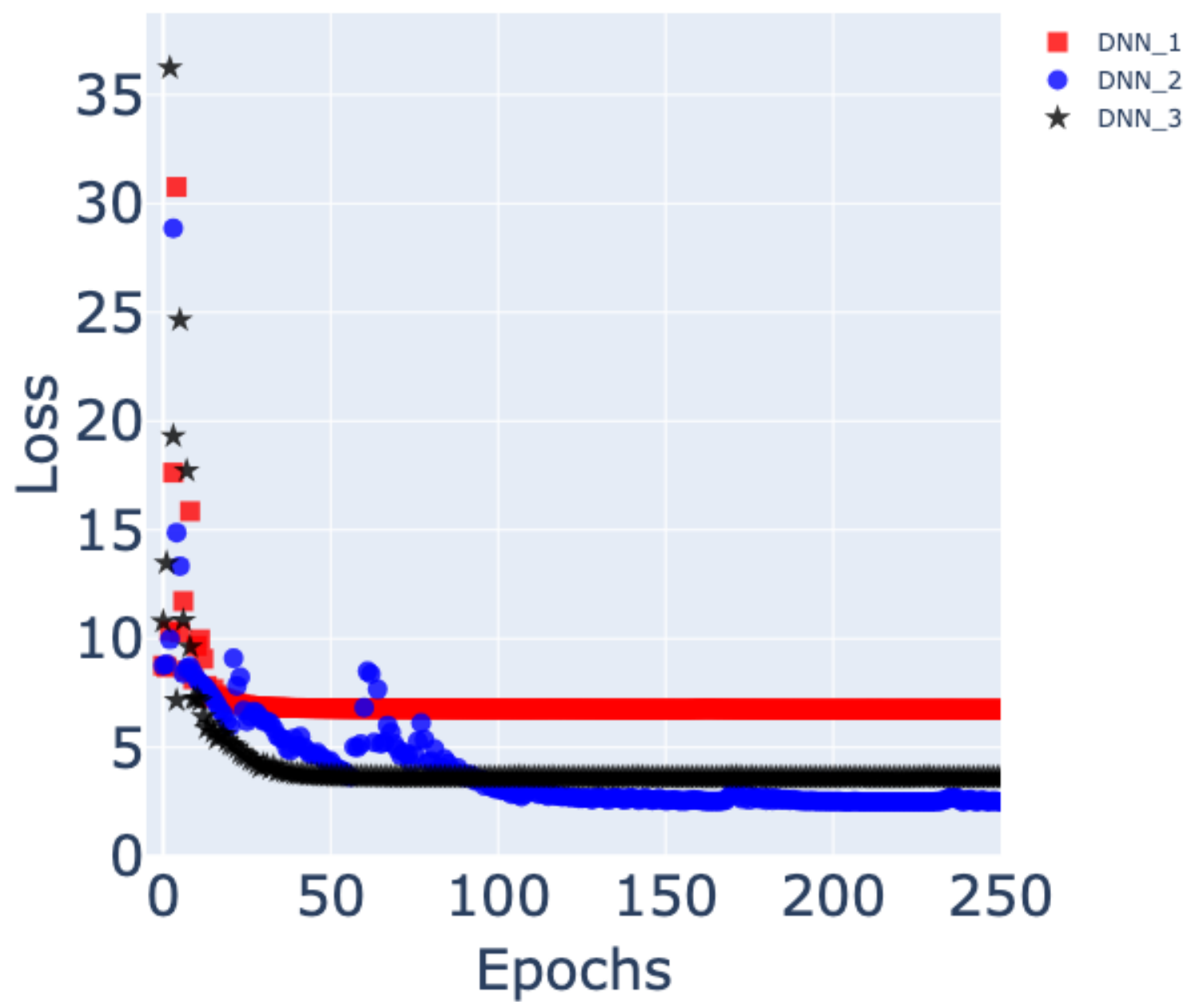

Figure S4. Training loss curves for the three deep neural network models. The red square markers, blue circular markers, and black star markers correspond to the loss curves of DNN_1, DNN_2, and DNN_3, respectively. Among the three models, DNN_2 achieves the best convergence, reaching a minimum loss of approximately 2.44, closely followed by DNN_3 with a minimum loss of about 3.56. In contrast, the basic architecture DNN_1 shows the poorest convergence behavior, with a substantially higher final loss of 6.73 throughout training. These results highlight the effectiveness of the additional features and architectural refinements introduced in DNN_2 and DNN_3 for improving model optimization and stability. The triangular mesh is obtained from CA1 dendritic reconstructions [25–27].

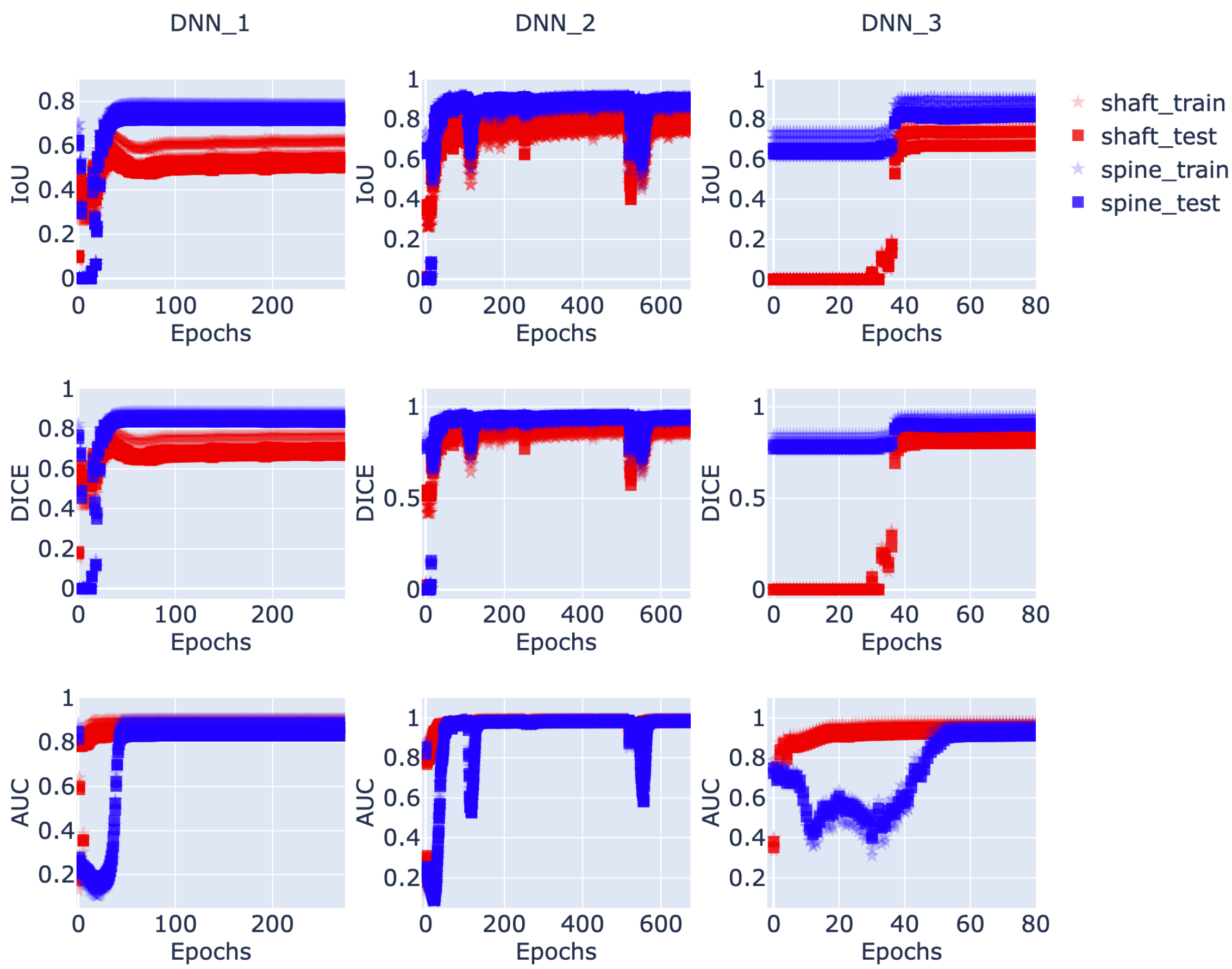

FIGURE S5. Performance curves for the three neural network models across the IoU, DICE, and AUC metrics. Columns 1, 2, and 3 correspond to DNN_1, DNN_2, and DNN_3, respectively, and each row reports a different evaluation metric. Red curves represent dendritic shaft predictions and blue curves represent spine predictions; star markers denote values computed on the *training* datasets, while square markers indicate the corresponding *validation* datasets, all derived from [25–27]. All curves are computed exclusively from the *non-augmented* data. Overall, DNN_2 and DNN_3 exhibit very similar performance, with average IoU values of approximately 0.70 (shaft) and 0.81 (spines), and average DICE values around 0.88 (shaft) and 0.92 (spines). In contrast, DNN_1 performs noticeably worse, particularly for shaft segmentation. AUC values remain high for all three models, with DNN_2 and DNN_3 again showing the strongest and most stable convergence. The close alignment between training and validation curves across all metrics indicates good generalization and minimal overfitting.

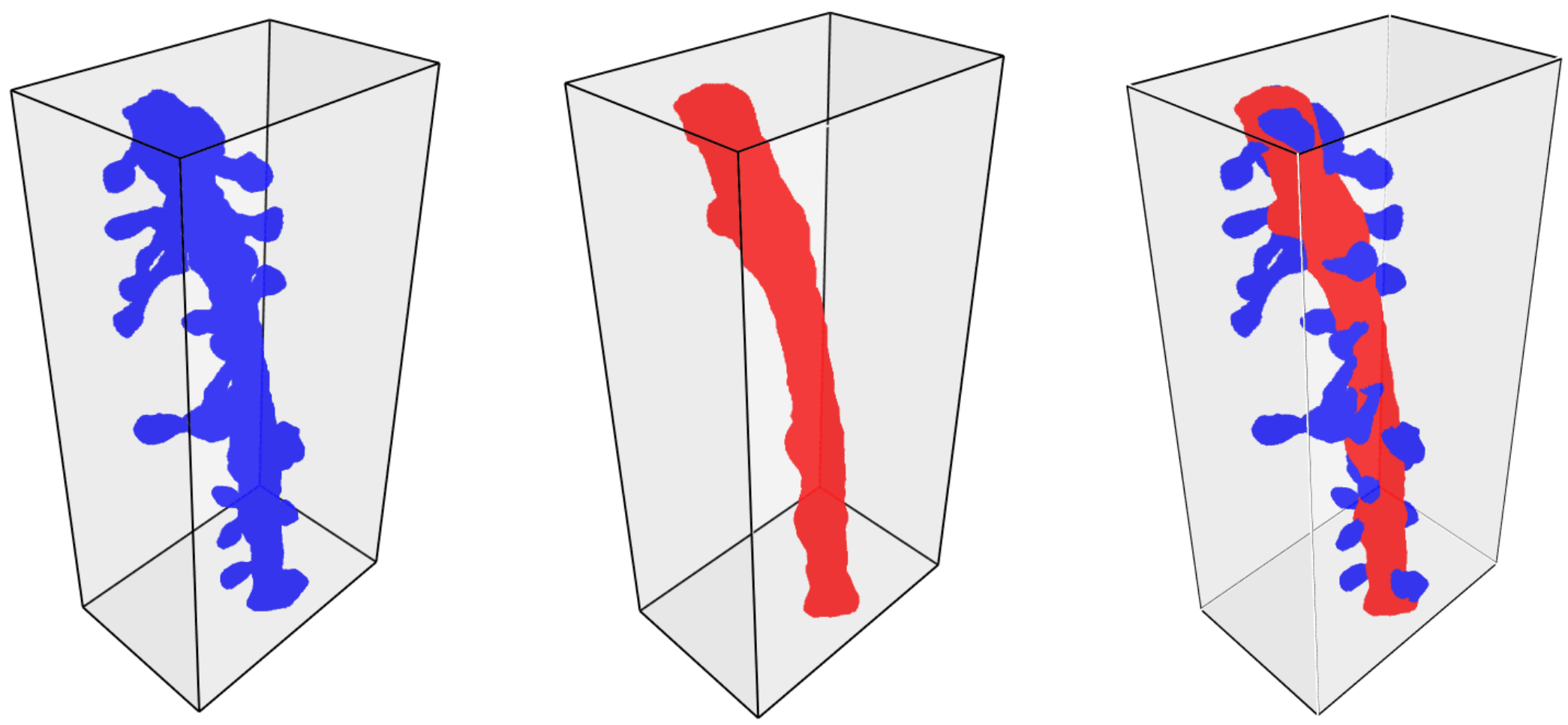

FIGURE S6. Construction of the voxel representations used for training. The top-left panel shows the voxelized triangular mesh of a dendritic branch (blue). The top-middle panel shows the ground-truth shaft labels generated by extending the annotated shaft mesh (red). The top-right panel shows the final target voxel grid, created by merging the dendrite-branch and shaft-label voxelizations and assigning the shaft label to overlapping voxels. This results in spine voxels labeled in blue and shaft voxels labeled in red. The bottom panel shows a slice view of the resulting volume in the plane defined by the two most informative PCA axes (PC1-PC2). The triangular mesh is obtained from CA1 dendritic reconstructions [25–27].

References

[1] Martín Abadi, Paul Barham, Jianmin Chen, Zhifeng Chen, Andy Davis, Jeffrey Dean, Matthieu Devin, Sanjay Ghemawat, Geoffrey Irving, Michael Isard, Manjunath Kudlur, Josh Levenberg, Rajat Monga, Sherry Moore, Derek G. Murray, Benoit Steiner, Paul Tucker, Vijay Vasudevan, Pete Warden, Martin Wicke, Yuan Yu, and Xiaoqiang Zheng. Tensorflow: a system for large-scale machine learning. In *Proceedings of the 12th USENIX Conference on Operating Systems Design and Implementation*, OSDI'16, page 265–283, USA, 2016. USENIX Association.

[2] Diederik P. Kingma and Jimmy Ba. Adam: A method for stochastic optimization. *CoRR*, abs/1412.6980, 2014.

[3] Robert Tibshirani. Regression shrinkage and selection via the lasso. *Journal of the royal statistical society series b-methodological*, 58:267–288, 1996.

[4] Arthur E Hoerl and Robert W Kennard. Ridge regression: Biased estimation for nonorthogonal problems. *Technometrics*, 12(1):55, February 1970.

[5] B. O'Neill. *Elementary Differential Geometry*. Academic Press, 1997.

[6] Mark Meyer, Mathieu Desbrun, Peter Schröder, and Alan H. Barr. Discrete differential-geometry operators for triangulated 2-manifolds. In Hans-Christian Hege and Konrad Polthier, editors, *Visualization and Mathematics III*, pages 35–57, Berlin, Heidelberg, 2003. Springer Berlin Heidelberg.

[7] John M Sullivan. Curvature measures for discrete surfaces. In *ACM SIGGRAPH 2006 Courses*, pages 10–13. 2006.

[8] Chen-Hung Wu, Thomas G. Fai, Paul J. Atzberger, and Charles S. Peskin. Simulation of osmotic swelling by the stochastic immersed boundary method. *SIAM Journal on Scientific Computing*, 37(4):B660–B688, 2015.

[9] Charles R Harris, K Jarrod Millman, Stéfan J van der Walt, Ralf Gommers, Pauli Virtanen, David Cournapeau, Eric Wieser, Julian Taylor, Sebastian Berg, Nathaniel J Smith, Robert Kern, Matti Picus, Stephan Hoyer, Marten H van Kerkwijk, Matthew Brett, Allan Haldane, Jaime Fernández Del Río, Mark Wiebe, Pearu Peterson, Pierre Gérard-Marchant, Kevin Sheppard, Tyler Reddy, Warren Weckesser, Hameer Abbasi, Christoph Gohlke, and Travis E Oliphant. Array programming with NumPy. *Nature*, 585(7825):357–362, September 2020.

[10] Qian-Yi Zhou, Jaesik Park, and Vladlen Koltun. Open3D: A modern library for 3D data processing. *arXiv:1801.09847*, 2018.

[11] Mike Dawson-Haggerty et al. Trimesh: A python library for loading and using triangular meshes. https://trimesh.org, 2019.

[12] Stéfan van der Walt, Johannes L Schönberger, Juan Nunez-Iglesias, François Boulogne, Joshua D Warner, Neil Yager, Emmanuelle Gouillart, Tony Yu, and scikit-image contributors. scikit-image: image processing in python. *PeerJ*, 2:e453, June 2014.

[13] Michael Kazhdan, Matthew Bolitho, and Hugues Hoppe. Poisson surface reconstruction. In *Proceedings of the Fourth Eurographics Symposium on Geometry Processing*, SGP '06, page 61–70, Goslar, DEU, 2006. Eurographics Association.

[14] Michael Kazhdan and Hugues Hoppe. Screened poisson surface reconstruction. *ACM Trans. Graph.*, 32(3), July 2013.

[15] Cem Yuksel. Sample elimination for generating poisson disk sample sets. *Comput. Graph. Forum*, 34(2):25–32, May 2015.

[16] H. Edelsbrunner, D. Kirkpatrick, and R. Seidel. On the shape of a set of points in the plane. *IEEE Transactions on Information Theory*, 29(4):551–559, 1983.

[17] Paolo Cignoni, Marco Callieri, Massimiliano Corsini, Matteo Dellepiane, Fabio Ganovelli, and Guido Ranzuglia. Meshlab: an open-source mesh processing tool. volume 1, pages 129–136, 01 2008.

[18] Michael Garland and Paul S. Heckbert. *Surface Simplification Using Quadric Error Metrics*. Association for Computing Machinery, New York, NY, USA, 1 edition, 2023.

[19] Pauli Virtanen, Ralf Gommers, Travis E. Oliphant, Matt Haberland, Tyler Reddy, David Cournapeau, Evgeni Burovski, Pearu Peterson, Warren Weckesser, Jonathan Bright, Stéfan J. van der Walt, Matthew Brett, Joshua Wilson, K. Jarrod Millman, Nikolay Mayorov, Andrew R. J. Nelson, Eric Jones, Robert Kern, Eric Larson, C. J. Carey, İlhan Polat, Yu Feng, Eric W. Moore, Jake VanderPlas, Denis Laxalde, Josef Perktold, Robert Cimrman, Ian Henriksen, E. A. Quintero, Charles R. Harris, Anne M. Archibald, Antônio H. Ribeiro, Fabian Pedregosa, Paul van Mulbregt, and SciPy 1. 0 Contributors. SciPy 1.0: fundamental algorithms for scientific computing in Python. *Nature Medicine*, 17:261–272, February 2020.

[20] P Dierckx. Algorithms for smoothing data with periodic and parametric splines. *Computer Graphics and Image Processing*, 20(2):171–184, 1982.

[21] Paul Dierckx. Curve and surface fitting with splines. In *Monographs on numerical analysis*, 1996.

[22] Özgün Çiçek, Ahmed Abdulkadir, Soeren S. Lienkamp, Thomas Brox, and Olaf Ronneberger. 3d u-net: Learning dense volumetric segmentation from sparse annotation. In Sebastien Ourselin, Leo Joskowicz, Mert R. Sabuncu, Gozde Unal, and William Wells, editors, *Medical Image Computing and Computer-Assisted Intervention – MICCAI 2016*, pages 424–432, Cham, 2016. Springer International Publishing.

[23] Wadha Al-Khater and Somaya Al-Madeed. Using 3d-vgg-16 and 3d-resnet-18 deep learning models and fabemd techniques in the detection of malware. *Alexandria Engineering Journal*, 89:39–52, 2024.

[24] Daniel Maturana and Sebastian Scherer. Voxnet: A 3d convolutional neural network for real-time object recognition. In *2015 IEEE/RSJ International Conference on Intelligent Robots and Systems (IROS)*, pages 922–928, 2015.

[25] Jennifer N Bourne and Kristen M Harris. Coordination of size and number of excitatory and inhibitory synapses results in a balanced structural plasticity along mature hippocampal ca1 dendrites during ltp. *Hippocampus*, 21(4):354–373, 2011.

[26] Cailey Bromer, Thomas M Bartol, Jared B Bowden, Dusten D Hubbard, Dakota C Hanka, Paola V Gonzalez, Masaaki Kuwajima, John M Mendenhall, Patrick H Parker, Wickliffe C Abraham, Terrence J

Sejnowski, and Kristen M Harris. Long-term potentiation expands information content of hippocampal dentate gyrus synapses. *Proc. Natl. Acad. Sci. U. S. A.*, 115(10):E2410–E2418, March 2018.

[27] Michael A Chirillo, Mikayla S Waters, Laurence F Lindsey, Jennifer N Bourne, and Kristen M Harris. Local resources of polyribosomes and ser promote synapse enlargement and spine clustering after long-term potentiation in adult rat hippocampus. *Scientific reports*, 9(1):3861, 2019.

[28] Jr Bartol, Thomas M, Cailey Bromer, Justin Kinney, Michael A Chirillo, Jennifer N Bourne, Kristen M Harris, and Terrence J Sejnowski. Nanoconnectomic upper bound on the variability of synaptic plasticity. *eLife*, 4:e10778, nov 2015.

[29] Narayanan Kasthuri, Kenneth Jeffrey Hayworth, Daniel Raimund Berger, Richard Lee Schalek, José Angel Conchello, Seymour Knowles-Barley, Dongil Lee, Amelio Vázquez-Reina, Verena Kaynig, Thouis Raymond Jones, Mike Roberts, Josh Lyskowski Morgan, Juan Carlos Tapia, H. Sebastian Seung, William Gray Roncal, Joshua Tzvi Vogelstein, Randal Burns, Daniel Lewis Sussman, Carey Eldin Priebe, Hanspeter Pfister, and Jeff William Lichtman. Saturated reconstruction of a volume of neocortex. *Cell*, 162(3):648–661, 2015.
